# Computational Identification of New Dual PAK4 and NAMPT Inhibitors

**DOI:** 10.3390/ijms27177706

**Published:** 2026-08-28

**Authors:** Yiling Wang, Audrey Minden

**Affiliations:** Susan Lehman Cullman Laboratory for Cancer Research, Department of Chemical Biology, Ernest Mario School of Pharmacy, Rutgers, The State University of New Jersey, Piscataway, NJ 08854, USA; yw798@pharmacy.rutgers.edu

**Keywords:** PAK4, NAMPT, dual inhibitors, molecular docking, cancer treatment

## Abstract

Dual inhibition of p21-activated kinase 4 (PAK4) and nicotinamide phosphoribosyltransferase (NAMPT) has emerged as a promising therapeutic strategy due to its ability to simultaneously target oncogenic signaling and cellular metabolism in cancer. While existing inhibitors such as KPT9274 and PF-3758309 have demonstrated preclinical activity, their clinical translation has been limited by poor selectivity, suboptimal efficacy, and dose-limiting toxicities. Reliance on a small number of available compounds is therefore insufficient, highlighting the need for systematic optimization to identify candidates with improved therapeutic profiles. Although several dual PAK4 and NAMPT inhibitors such as GNE2861, LCH7749944, and PF3758309 have been identified in our previous study, the number of compounds available is limited. Identification of new candidate compounds would allow researchers to identify those that are the most efficient, those that are the most potent and selective, and those that have optimal pharmacokinetics. In this study, we performed additional large-scale drug screening to identify new candidate dual PAK4 and NAMPT inhibitors and expand the diversity of this inhibitor class. Computational molecular docking was used to evaluate binding affinities toward both targets, followed by drug–protein interaction analyses to assess binding stability and interaction patterns at the molecular level. Several new candidate compounds demonstrated favorable predicted binding to both PAK4 and NAMPT, with distinct interaction profiles compared to previously reported inhibitors. Biochemical assays further demonstrated inhibitory activity against both PAK4 and NAMPT among several selected compounds, supporting their potential as dual-target inhibitors. These findings provide mechanistic insight into dual-target engagement and highlight structural features associated with improved binding behavior. Expanding the pool of dual inhibitors enhances opportunities for preclinical development, supports optimization of pharmacokinetic and safety profiles, and strengthens datasets for drug discovery. Collectively, this work broadens the landscape of dual PAK4 and NAMPT inhibitors and supports their potential application across multiple cancer types beyond triple-negative breast cancer.

## 1. Introduction

The p21-activated kinases (PAKs) are a family of serine/threonine kinases that act as effectors of the small GTPases Cdc42 and Rac1, regulating essential cellular processes such as proliferation, survival, cytoskeletal organization, and motility [1,2,3,4]. They are divided into two groups based on structural and functional differences: group I (PAK1–3) and group II (PAK4–6) [5,6,7,8,9]. Among them, PAK4, a member of group II, plays a central role in controlling cell growth, migration, and invasion [10,11,12,13,14]. Dysregulation or overactivation of PAK4 has been observed in multiple cancers, including breast, ovarian, pancreatic, and colorectal cancers, where activated or overexpressed PAK4 promotes tumor progression, enhances cellular adaptation to stress, and plays a key role in metastatic spread [15,16,17,18,19]. PAK4 exerts these effects by modulating downstream signaling pathways, reorganizing the cytoskeleton, inhibiting apoptosis, and modulating the tumor microenvironment and immunity [19,20,21,22]. Its role in these processes makes PAK4 a promising target for developing anticancer therapies.

NAMPT is the rate-limiting enzyme in the NAD^+^ (nicotinamide adenine dinucleotide) salvage pathway, which recycles nicotinamide to produce NAD^+^, an essential cofactor for energy metabolism, redox homeostasis, and DNA repair [23,24,25]. High NAD^+^ levels are essential for the rapid proliferation of cancer cells. NAMPT overexpression has been reported in numerous cancers and correlates with poor prognosis, increased tumor growth, and resistance to some types of therapy [26,27,28]. Pharmacological inhibition of NAMPT disrupts NAD^+^ and NADP^+^ (Nicotinamide adenine dinucleotide phosphate) metabolism, leading to metabolic stress, accumulation of reactive oxygen species, and cell death [26,29,30]. Because NAD^+^ metabolism is central to multiple cellular processes, NAMPT represents a highly attractive target for cancer treatment, particularly for tumors with elevated metabolic demands [27,28,31,32,33].

Targeting both PAK4 and NAMPT simultaneously offers a strategy to interfere with oncogenic signaling and cellular metabolism at the same time, potentially enhancing anticancer efficacy [14,34]. However, the current pool of dual PAK4 and NAMPT inhibitors is limited, restricting the ability to optimize compounds that are the most potent and selective and have favorable pharmacokinetic properties [35]. PF-3758309, an early PAK4 inhibitor, advanced into Phase I clinical development but was discontinued due to poor selectivity, adverse events, and unfavorable pharmacokinetic properties [10,36,37]. KPT-9274, an allosteric dual inhibitor targeting both PAK4 and NAMPT, has demonstrated promising anticancer activity in multiple cancer types [14,38,39,40]. Compared to PF-3758309, KPT-9274 is more selective but showed no objective tumor responses in Phase I and is limited by dose-dependent toxicities, including kidney and stomach injury, anemia, and gender-specific effects on erythropoiesis observed in mouse preclinical models [39,41,42]. Similarly, first-generation NAMPT inhibitors, such as FK866 and CHS828, showed limited efficacy in the clinic [43,44,45]. Some of them exhibited severe toxicity, which limits their usable dosage, and some serious side effects, including thrombocytopenia, gastrointestinal, and retinal toxicities [42,46]. These findings highlight the need for additional dual inhibitors with improved selectivity, efficacy, and safety profiles. Identification of a larger, more diverse pool of dual inhibitors could improve therapeutic potential and allow their use in treating multiple cancer types.

Previous studies [38] have shown that several PAK4 inhibitors, such as KPT-9274, PF-3758309, GNE2861, and LCH7749944, not only suppress PAK4 activity but also inhibit NAMPT, thereby decreasing NAD^+^ and NADP^+^ levels in cancer cells [15,18,38,47,48,49,50]. This dual activity provides a unique opportunity to simultaneously target oncogenic signaling and metabolic pathways [14,34,40]. However, the clinical translation of existing dual inhibitors has been limited by suboptimal efficacy, poor selectivity, and dose-limiting toxicities [31,35,42,51]. Furthermore, the limited number of available compounds restricts optimization of potency, pharmacokinetic properties, and safety; therefore, there is a need for additional dual inhibitors with improved therapeutic potential.

**Figure 1:**
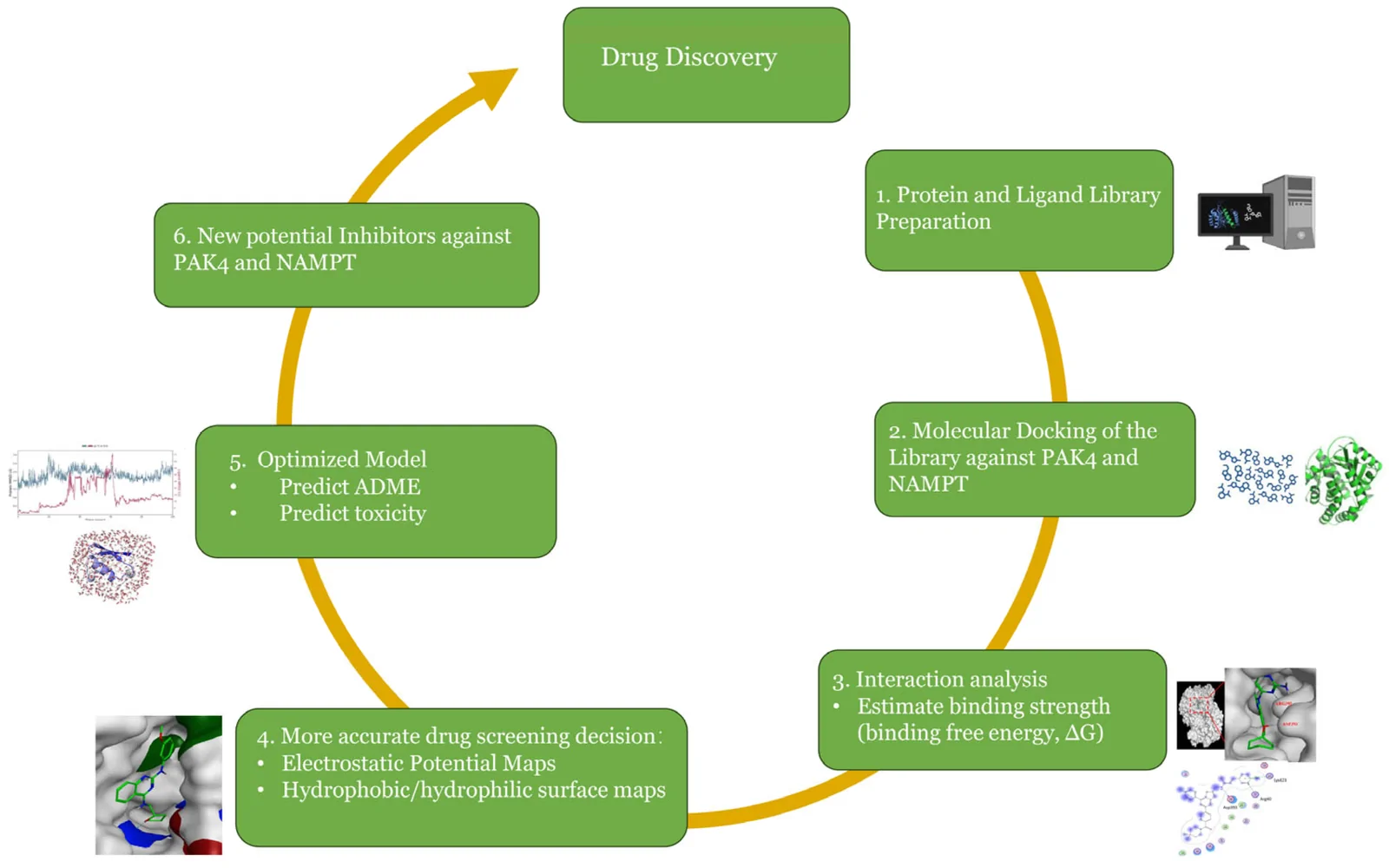
Schematic workflow for virtual screening in drug discovery. The procedure includes protein and ligand preparation, molecular docking against PAK4 and NAMPT, interaction and binding energy analysis, electrostatic and surface property evaluation, prediction of ADME (absorption, distribution, metabolism, and excretion) and toxicity, leading to identification of new potential PAK4 and NAMPT inhibitors.

Due to these limitations of existing dual PAK4/NAMPT inhibitors, we performed large-scale drug screening to identify novel candidates. To enrich the chemical space for potential anticancer candidates, we selected a curated PubChem library of 1173 compounds with reported anticancer activity for virtual screening against PAK4 and NAMPT. These compounds were evaluated using computational molecular docking, and drug–protein interaction analyses to assess predicted binding affinity, stability, and interactions at the molecular level [9,52,53,54,55]. Expanding the chemical diversity of dual inhibitors leads to additional options for disease treatment and also strengthens datasets that can be used for other drug discovery studies. The overall virtual screening workflow is illustrated in Figure 1. By combining prior docking data with virtual screening, Absorption, Distribution, Metabolism, Excretion (ADME) and toxicity predictions, we aim to uncover compounds with previously unrecognized activity against PAK4, NAMPT, or both, for preclinical evaluation. Beyond the potential applications in breast cancer, this strategy establishes a framework for drug repurposing and the exploration of dual or selective inhibition in other cancers and diseases. Overall, the goal of this approach is to identify new therapeutic candidates and provide a platform for identifying new drugs for use in oncology and other disease areas.

## 2. Results

### 2.1. Molecular Docking

**Figure 2:**
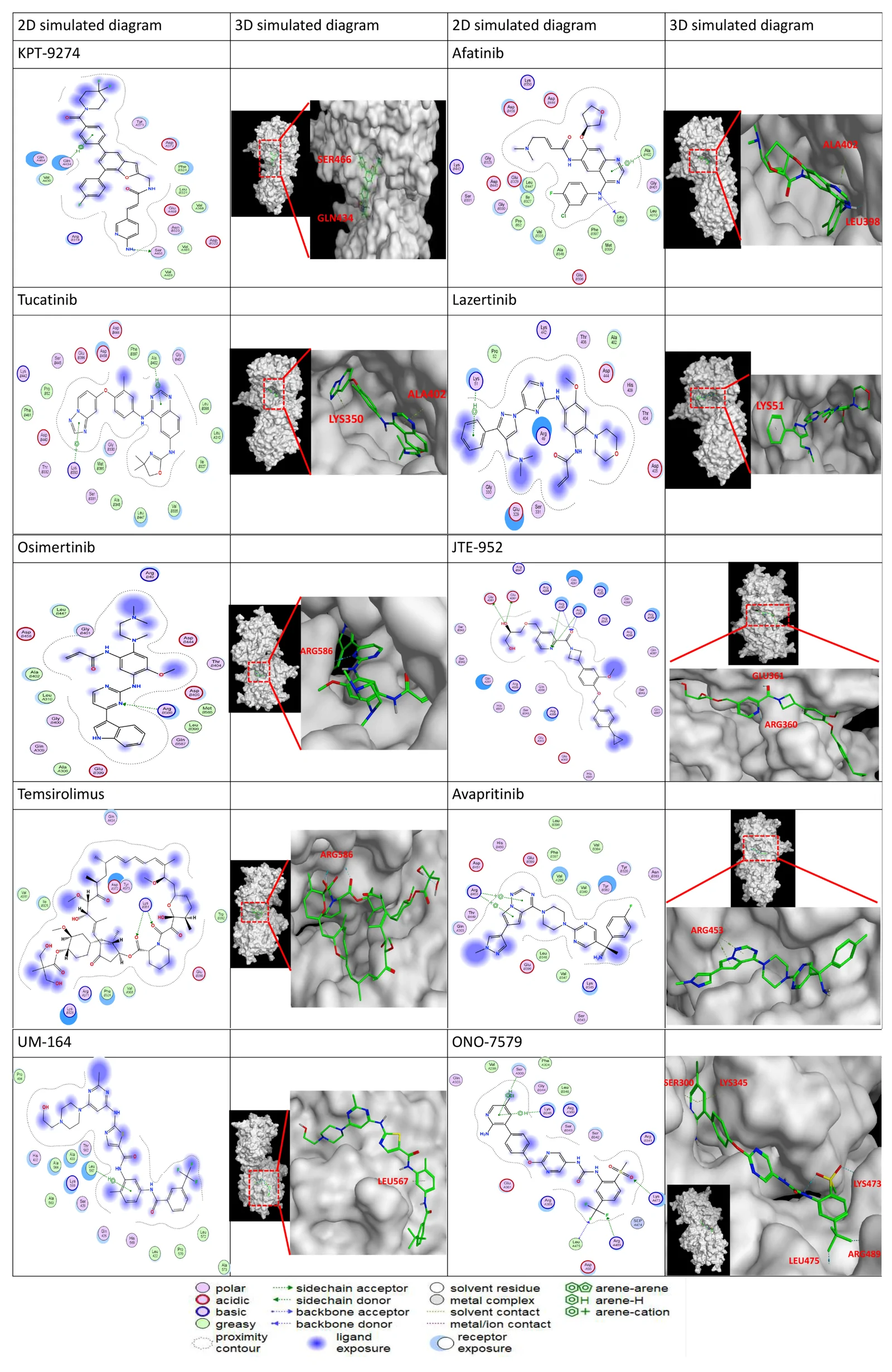
The predicted interactions between top candidate compounds and the PAK4 protein. 2D simulated binding diagrams illustrate the interaction patterns, including hydrogen bonds, hydrophobic interactions, and amino acid residues involved in ligand binding within the PAK4 active site. 3D simulated binding diagrams show the binding orientations of the candidate compounds within the PAK4 binding pocket. The protein is displayed as a ribbon model, and the compounds are shown as stick representations. Key interacting residues are highlighted to demonstrate the predicted interactions and orientation of each ligand within the binding site.

Large-scale virtual screening was performed to identify novel compounds predicted to bind both PAK4 and NAMPT. Molecular docking results showed that multiple candidates exhibited favorable binding affinities toward both targets, comparable to those of the previously reported dual inhibitor KPT9274. Several compounds demonstrated strong predicted binding to the ATP-binding pocket of PAK4 (See Figure 2), forming key interactions with residues involved in kinase activity, including hydrogen bonding with hinge-region residues and hydrophobic contacts within the catalytic cleft.

**Figure 3:**
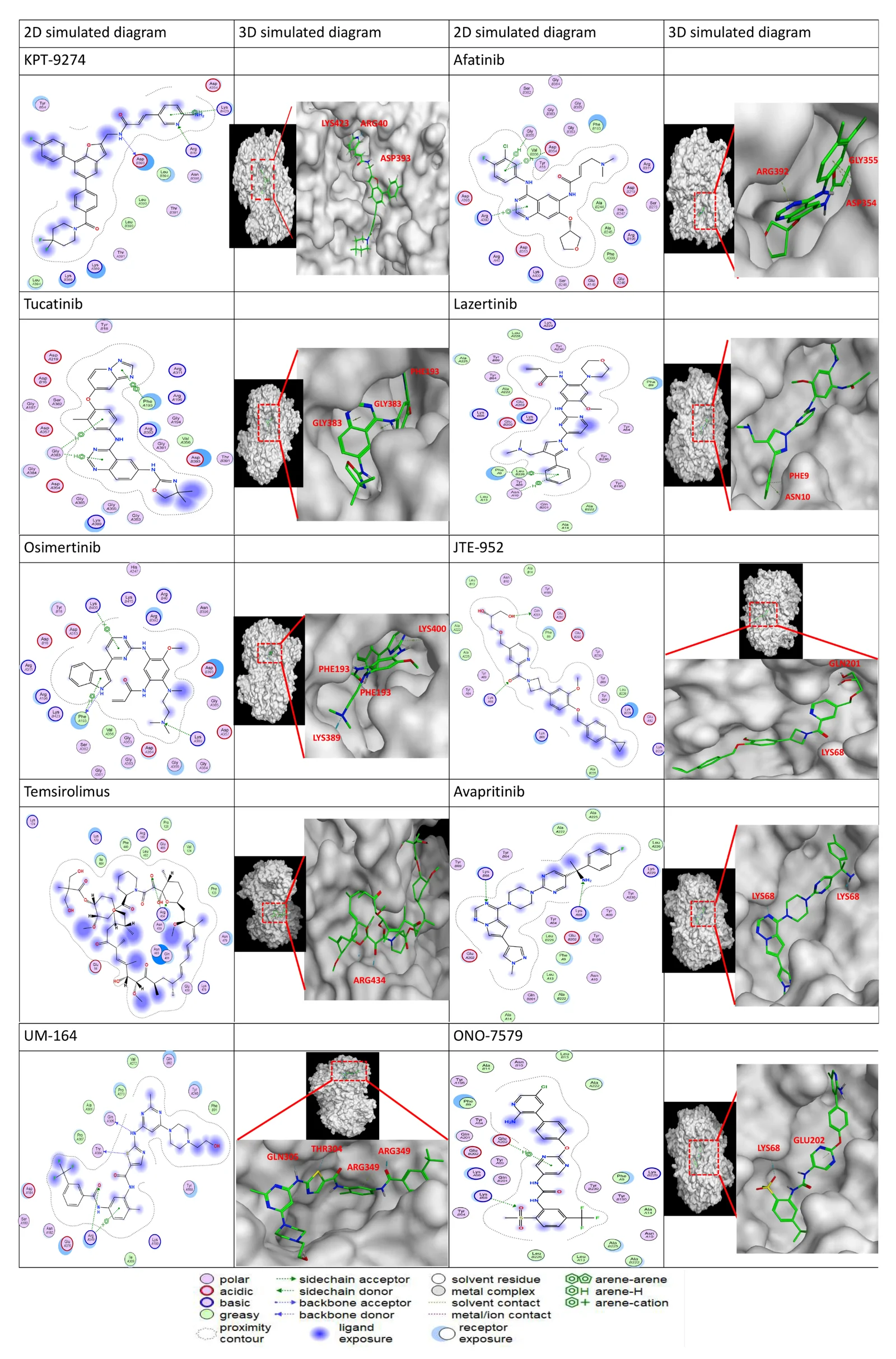
The predicted interactions between top candidate compounds and the NAMPT protein. 2D simulated binding diagrams illustrate the interaction patterns, including hydrogen bonds, hydrophobic interactions, and amino acid residues involved in ligand binding within the NAMPT active site. 3D simulated binding diagrams show the binding orientations of the candidate compounds within the NAMPT binding pocket. The protein is displayed as a ribbon model, and the compounds are shown as stick representations. Key interacting residues are highlighted to demonstrate the stability and orientation of each ligand within the binding site.

Docking against NAMPT revealed that selected candidates were predicted to occupy the nicotinamide-binding site (See Figure 3), forming stabilizing interactions with residues critical for enzymatic activity. These predicted interactions included hydrogen bonds with catalytic residues and hydrophobic contacts that are known to contribute to NAMPT inhibition (See Table 1 and Table 2). Table 1 and Table 2 show KPT-9274 and only the top 10 candidate compounds; the top 50 compounds are in the Supplementary Materials (Table S1). Importantly, several compounds displayed distinct binding poses compared to known dual inhibitors, suggesting alternative interaction patterns. These predicted interaction mechanisms may contribute to improved selectivity or binding efficiency. Overall, docking results indicate that the newly identified compounds have properties consistent with dual inhibition of PAK4 and NAMPT.

### 2.2. ADME Property Prediction

ADME prediction was performed to evaluate the drug-like properties of the selected dual inhibitors. Most compounds showed favorable molecular weight, lipophilicity (LogP), and topological polar surface area (TPSA), along with high predicted intestinal absorption and better solubility. The molecular weights of compounds are within 480–650 g/mol, while only Temsirolimus showed a markedly higher value at 1030.29 g/mol. TPSA values ranged from 88.61 to 241.96 Å^2^, with the majority clustering around 90–110 Å^2^, whereas Temsirolimus exhibited substantially higher polarity. Lipophilicity (consensus Log P) varied from 2.22 to 5.86, indicating differences in hydrophobicity across the dataset. Compared with KPT-9274, several candidates demonstrated better predicted aqueous solubility, which may further enhance absorption. High gastrointestinal absorption was predicted for most candidates, except KPT-9274, Temsirolimus, UM164 and ONO-7579, which showed low GI absorption. Pharmacokinetic analysis suggested limited blood–brain barrier permeability for most compounds, indicating reduced likelihood of central nervous system-related side effects. However, most compounds were predicted to be P-glycoprotein substrates, which may influence efflux and bioavailability. CYP450 inhibition profiling indicated that most candidates acted as inhibitors of major drug-metabolizing enzymes, including CYP2C19, CYP2C9, CYP2D6, and CYP3A4. These enzymes play key roles in hepatic drug metabolism, and their inhibition may alter the clearance of co-administered drugs, suggesting potential metabolic liability and drug–drug interaction risk [56,57]. Afatinib, Tucatinib, Lazertinib, Osimertinib, JTE-952 and ONO-7579 showed broad inhibition across these CYP isoforms, whereas KPT-9274, Temsirolimus, Avapritinib, and UM164 exhibited comparatively fewer CYP interactions. Most compounds may present a high likelihood of metabolic liability due to inhibition of key hepatic drug clearance pathways. Overall, these results indicate that while the selected compounds exhibit favorable absorption and drug-like characteristics, further optimization may be required to improve metabolic stability and minimize CYP-mediated interactions (see Table 3). This is important because it may reduce the risk of adverse effects that can be associated with the central nervous system. Overall, ADME predictions indicate that several newly identified compounds possess improved pharmacokinetic properties suitable for further preclinical evaluation.

### 2.3. Toxicity Prediction

Computational toxicity analysis was used to predict the toxicity and safety profiles of selected dual inhibitors. Most compounds showed low predicted risk for mutagenicity, cardiotoxicity, and hepatotoxicity, compared with KPT-9274. Predicted oral toxicity scores ranged from 3 to 5, with KPT-9274 scoring 4. Tucatinib, UM-164, Temsirolimus, and Avapritinib scored 5, indicating lower predicted toxicity than KPT-9274, while Osimertinib scored 3, suggesting higher risk. Organ toxicity endpoints were predicted as inactive (no or low risk) for most compounds, although neurotoxicity and respiratory toxicity were predicted as active (high risk) for all candidates. Carcinogenicity was predicted for all candidates, whereas mutagenicity and immunotoxicity were mostly inactive. Tox21 pathway analysis was used to provide mechanistic insight into toxicity risks by linking compounds to nuclear receptor and stress response pathways associated with regulatory disruption involving hormone signaling and gene transcription networks [58,59]. Most compounds were predicted to show no significant interaction (“inactive” in Table 4) with key nuclear receptors, including AhR, AR-LBD, aromatase, ER, ER-LBD, and PPAR-γ, indicating limited involvement in these signaling pathways. Interaction (“active” in Table 4) with the androgen receptor (AR) and Nrf2/ARE pathways was observed for JTE-952 and Temsirolimus, suggesting potential involvement in oxidative stress and hormone-related signaling. Molecular initiating events describe the earliest molecular interactions that trigger toxicity cascades and provide mechanistic insight into downstream adverse outcomes. Most targets were predicted to be inactive across the compound set. However, selective activation was observed for THRα, TTR, GABAR, and PXR, particularly for Lazertinib. In addition, NMDAR interactions were predicted for Afatinib, Tucatinib, Lazertinib, ONO-7579, and UM164, indicating potential effects on neuronal signaling pathways. Overall, compared with KPT-9274, most compounds exhibited comparable or more favorable predicted toxicity profiles in specific endpoints, including oral toxicity class and selected organ toxicity parameters, although shared potential adverse effects such as neurotoxicity, respiratory toxicity, and carcinogenicity were observed across all candidates. While in silico predictions require experimental validation, these results suggest that a wider pool of dual inhibitors may be advantageous and enable the identification of more effective and safer compounds.

### 2.4. Molecular Electrostatic Potential (MEP)

**Figure 4:**
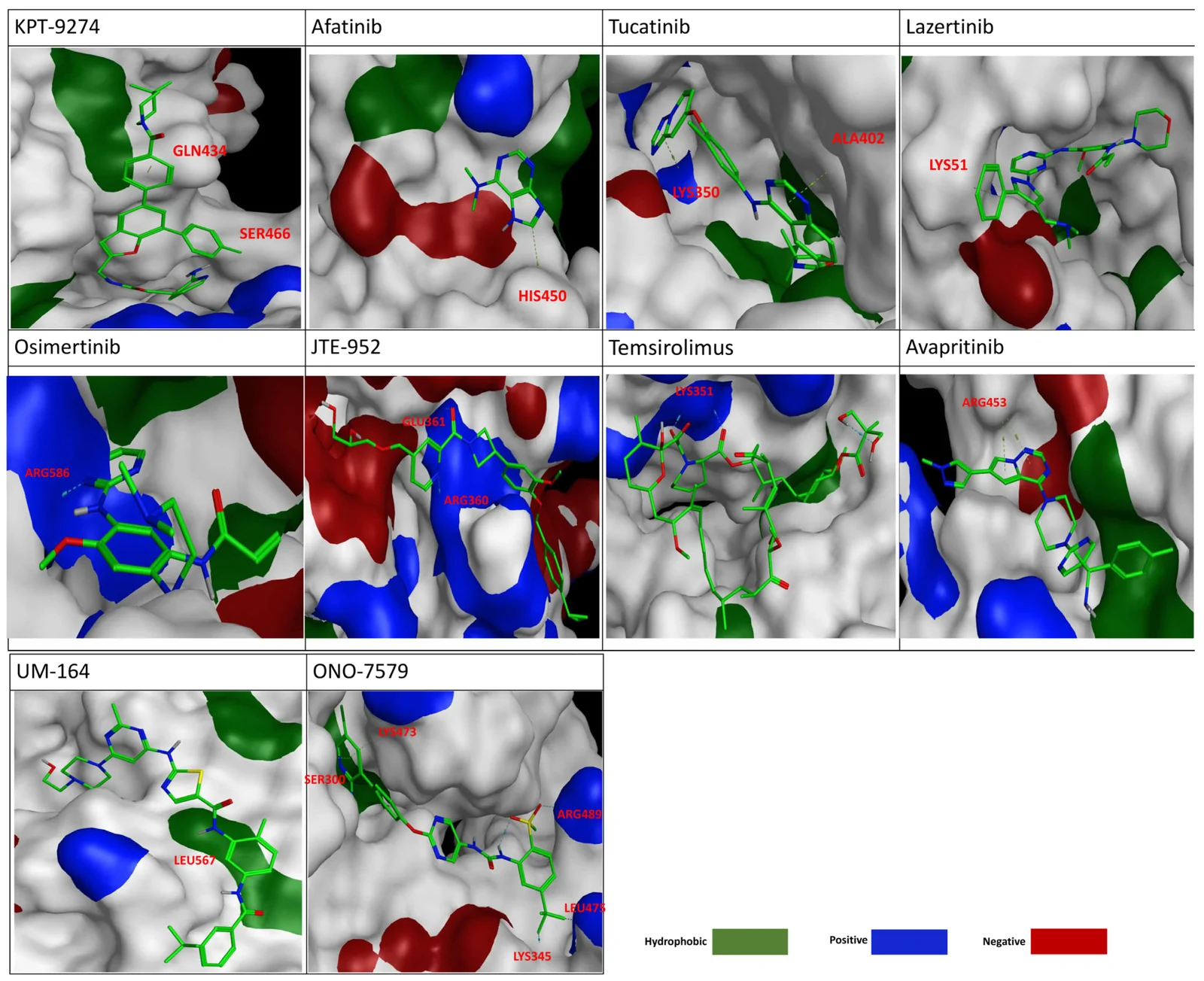
Molecular electrostatic potential (MEP) maps of the top candidate compounds in complex with the PAK4 protein. Hydrophobic regions are green, positively charged in blue, and negatively charged in red. KPT-9274 shows mainly hydrophobic surfaces with localized charges and π-based interactions. The top candidates display more distributed charged regions, interact with positively charged residues, and form π-based interactions within the ATP-binding pocket, highlighting key binding interactions that contribute to their predicted affinity for PAK4.

**Figure 5:**
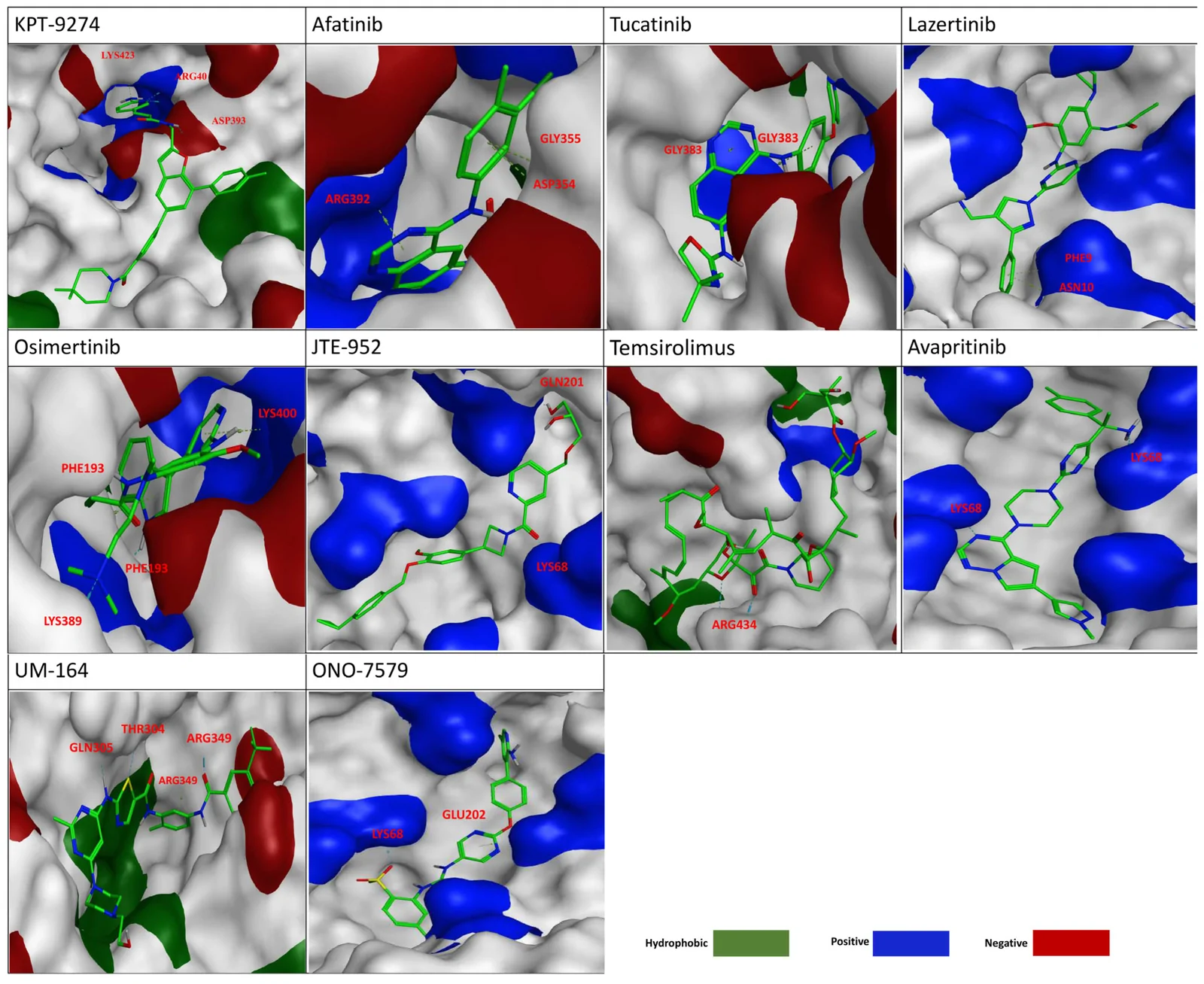
The MEP map of top candidate compounds in complex with the NAMPT Protein. Hydrophobic regions are green, positively charged in blue, and negatively charged in red. KPT-9274 shows mainly hydrophobic surfaces with localized charges and π-based interactions. The top candidates display more distributed positive and negative regions along with π-based interactions, which may contribute to their higher predicted affinity for NAMPT.

Molecular electrostatic potential (MEP) surfaces were generated for the selected top-ranked dual PAK4 and NAMPT inhibitors to characterize their predicted charge distribution (hydrophobic (green), positive (blue), and negative (red) at the ligand–protein interface (See Figure 4 and Figure 5). For the MEP at PAK4, KPT-9274 was predicted to be primarily surrounded by a continuous hydrophobic surface, with positive and negative potentials appearing as relatively localized patches, and it mainly formed π-based non-electrostatic interactions. In contrast, the predictions indicate that the top candidates exhibited a more spatially distributed arrangement of positive and negative potential regions around the ligand, which interacted with key positively charged residues, including HIS450, LYS350, LYS51, ARG586, ARG360, and ARG453, in addition to forming π-based interactions. Similarly, at NAMPT, KPT-9274 was surrounded by hydrophobic surfaces with localized charged patches, forming π-based interactions with residues such as ASP393 and ARG40. The top candidates displayed more distributed charged regions, particularly near positively charged residues LYS68, ARG434, and ARG392, while also engaging in π-based interactions. These compounds showed enhanced polarity at functionally relevant sites, but also maintained electrostatic balance. The MEP profiles of the top candidates were consistent with their predicted dual binding behavior, and they were selected for further ADME evaluation and potential toxicity.

### 2.5. NAMPT Activity Assay

**Figure 6:**
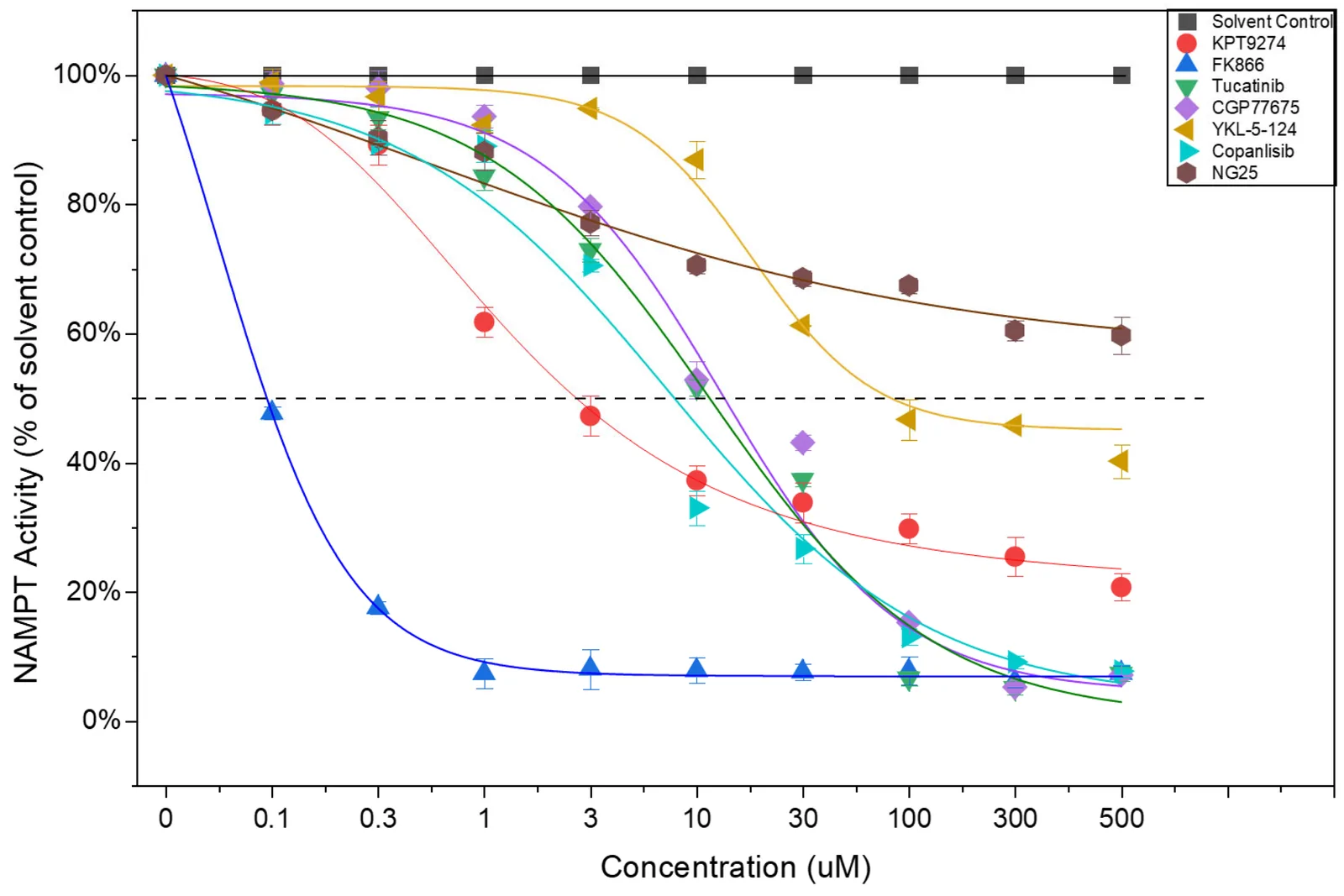
Several top candidate compounds inhibit NAMPT activity in a cell-free assay. NAMPT activity in response to top candidate compounds (Tucatinib, CGP77675, YKL-5-124, Copanlisib, and NG25), dual PAK4/NAMPT inhibitors (KPT-9274), the solvent control (DMSO), and FK866, the positive control.

To experimentally assess the virtual screening results, several top-ranked compounds were selected from the top 50 candidate dual PAK4/NAMPT inhibitors identified through the computational screening workflow and evaluated in a recombinant NAMPT cell-free assay. PF-3758309 and FK866 were included as positive controls, while Phloretin served as a negative control. In addition, KPT-9274, a known dual PAK4/NAMPT inhibitor, was included as a reference compound. NAMPT activity in vehicle-treated samples (DMSO) was defined as 100% (See Figure 6). As expected, FK866 produced near-complete inhibition of NAMPT activity, reducing residual enzyme activity to 4.68%. PF-3758309 also demonstrated substantial NAMPT inhibition, with residual activity of 16.80% at 100 μM, whereas Phloretin showed no inhibitory effect. Among the screened candidates, CGP77675 and Copanlisib exhibited the strongest inhibitory effects on NAMPT, reducing enzyme activity to 5.24% and 9.15%, respectively, producing an extent inhibition comparable to that of FK866. KPT-9274 also showed strong NAMPT inhibition, decreasing residual activity to 29.77%. YKL-5-124 and Tucatinib displayed moderate inhibitory activity, reducing NAMPT activity to 45.22% and 56.55%, respectively, while NG25 showed relatively weak inhibition with 57.44% residual activity. In contrast, Lazertinib, UM-164, Adavosertib, GSK2636771, and Everolimus produced little or no inhibition of NAMPT activity, with residual enzyme activities remaining between 90% and 95% of the vehicle control. Based on these results, CGP77675, Copanlisib, Tucatinib, YKL-5-124, and NG25 were selected for further PAK4 kinase activity. Dose–response studies confirmed NAMPT inhibition, with KPT-9274 showing an IC_50_ of 4.22 μM. Among the screened compounds, Copanlisib was the most potent (5.8 μM), followed by Tucatinib (11.4 μM) and CGP77675 (13.8 μM), while YKL-5-124 and NG25 showed weaker activity. Overall, Copanlisib, Tucatinib, and CGP77675 emerged as the most promising NAMPT inhibitors among the screened compounds, supporting their further investigation as potential dual PAK4/NAMPT inhibitors.

### 2.6. PAK4 Activity Assay

**Figure 7:**
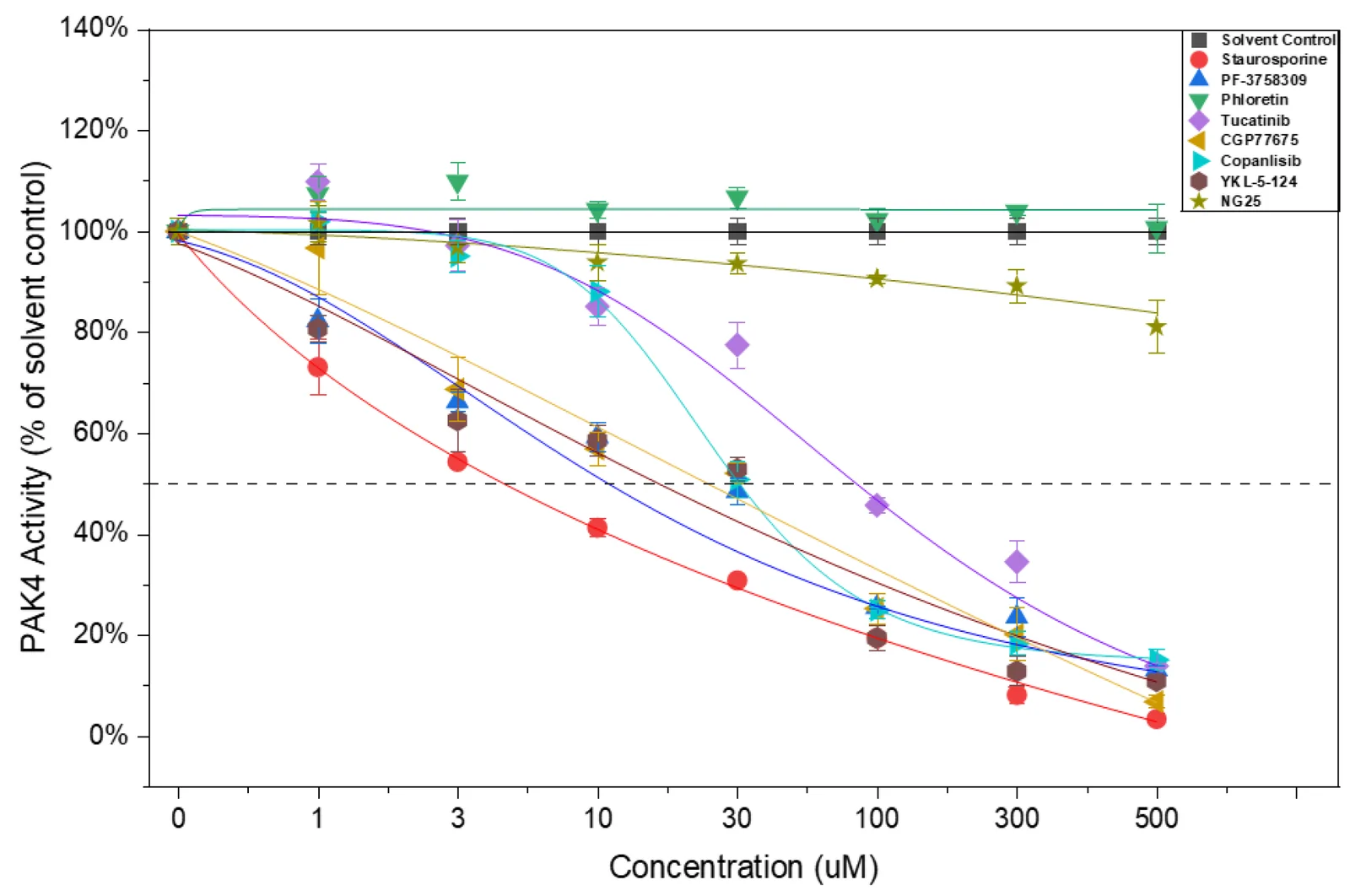
Several top candidate compounds inhibit PAK4 activity in a cell-free assay. PAK4 activity in response to top candidate compounds (Tucatinib, CGP77675, YKL-5-124, Copanlisib, and NG25), dual PAK4/NAMPT inhibitors (KPT-9274), the solvent control (DMSO), and Staurosporine, the manufacturer-recommended positive control.

To assess the PAK4 inhibitory activity of the selected compounds, candidates showing promising NAMPT inhibition were further tested using a recombinant PAK4 kinase assay. The effects of the selected compounds on PAK4 activity are shown in Figure 7. Staurosporine, a broad-spectrum protein kinase inhibitor recommended by the manufacturer as a positive control for the PAK4 kinase assay, was included as the positive control. Phloretin, a naturally occurring flavonoid with reported anti-inflammatory and other biological activities, but not a known PAK4 kinase inhibitor, was used as the negative control. Initial screening at 100 μM showed that staurosporine and PF-3758309 strongly inhibited PAK4 activity, reducing residual kinase activity to 14.73% and 13.46%, respectively, whereas phloretin showed little or no inhibition (92.27% residual activity). Among the compounds selected from the top 50 computationally identified candidates, CGP77675, Copanlisib, and YKL-5-124 exhibited strong PAK4 inhibition, reducing residual kinase activity to 14.36%, 12.90%, and 13.16%, respectively, while Tucatinib also demonstrated substantial inhibition with 42.99% residual activity. In contrast, NG25 showed little inhibitory activity (92.13% residual activity). KPT-9274 did not inhibit PAK4 activity in this ATP-dependent assay format, consistent with its reported allosteric mechanism of PAK4 inhibition. Based on these results, CGP77675, Copanlisib, YKL-5-124, and Tucatinib were selected for dose–response studies. IC_50_ analysis revealed PAK4 inhibition by YKL-5-124 (4.10 μM), CGP77675 (4.79 μM), Copanlisib (5.02 μM), and Tucatinib (5.86 μM). Together with the NAMPT activity results, CGP77675, Copanlisib, Tucatinib, and YKL-5-124 showed inhibitory activity against both PAK4 and NAMPT, whereas NG25 inhibited NAMPT but showed little PAK4 inhibition. These findings identify CGP77675 and Copanlisib as the most promising dual PAK4/NAMPT inhibitors among the compounds evaluated.

## 3. Discussion

In the present study, large-scale virtual screening, combined with molecular docking, ADME predictions, toxicity evaluation, and electrostatic potential analysis, was performed to identify novel dual inhibitors targeting both PAK4 and NAMPT. Dual inhibition of these two proteins has attracted increasing attention because PAK4 regulates cell survival signaling, while NAMPT controls NAD^+^ biosynthesis and cellular metabolism [23,26,27,60]. Simultaneous targeting of these pathways may improve therapeutic efficacy compared with single-target inhibitors [14,38,61,62]. The currently reported dual inhibitor KPT-9274 demonstrates the feasibility of this strategy, but its pharmacokinetic limitations and toxicity concerns highlight the need to identify additional compounds with improved properties [14,39]. The results of this study expand the number of potential dual inhibitors and provide several promising candidates for further experimental testing.

Molecular docking results showed that multiple newly identified compounds exhibited predicted binding affinities for PAK4 comparable to or stronger than those of KPT9274. The top candidates formed key hydrogen bonds with residues in the hinge region of the kinase domain and established hydrophobic contacts within the ATP-binding pocket. These interactions are consistent with known requirements for effective kinase inhibition and suggest that the selected compounds may efficiently block PAK4 catalytic activity [2,5,10]. In addition to conserved interactions, several compounds displayed alternative binding orientations. This may contribute to improved selectivity, or it can help reduce off-target effects. The ability to bind the ATP pocket through different interaction patterns may help avoid mutations that reduce inhibitor binding.

Docking against NAMPT further demonstrated that the selected compounds can occupy the nicotinamide-binding site and form stabilizing interactions with catalytic residues critical for enzymatic activity. Hydrogen bonding with conserved residues and hydrophobic contacts within the active site were observed for most candidates, supporting their potential to inhibit NAD^+^ biosynthesis. Interestingly, several compounds showed stronger predicted binding scores for NAMPT than KPT9274, suggesting that improved inhibition of metabolic pathways may be achievable with these molecules. Because NAMPT inhibition is often associated with toxicity, identifying compounds that bind both targets in a balanced manner may be important for achieving the most effective dual inhibition.

Molecular dynamics simulations were performed to validate docking results under dynamic conditions and to evaluate the stability of ligand–protein complexes [52,54,63,64]. Most complexes reached equilibrium early during the simulation and remained stable, indicating favorable binding stability. RMSF analysis showed limited fluctuations in ligand positions within the binding pockets, suggesting that the interactions predicted by docking were maintained during the simulation. In several cases, additional transient hydrogen bonds were observed, which may further stabilize the complexes. These results support the reliability of the docking predictions and indicate that the selected compounds can form stable interactions with both PAK4 and NAMPT in a dynamic environment.

Molecular electrostatic potential (MEP) analysis provided additional insight into the molecular features responsible for dual binding. The MEP maps showed clear separation of positive and negative electrostatic regions, allowing electrostatic interactions within the binding pockets. Compounds with strong docking scores typically displayed negative potential near carbonyl or heteroatom groups positioned toward hydrogen bond donor residues, while positive potential regions were oriented toward negatively charged amino acids. Compared with KPT-9274, several new candidates showed a more balanced electrostatic distribution, which may improve binding efficiency and reduce unfavorable interactions. These observations suggest that electrostatic complementarity plays an important role in achieving dual-target engagement.

Combining molecular docking and MEP results, the ligands were predicted to be in close proximity to the protein, forming non-covalent contacts, including π–π stacking and van der Waals interactions. Van der Waals contacts are inferred from these close contacts, while π–H (2.5–3.0 Å) and π–cation (3.0–6.0 Å) interactions are specific aromatic interactions that stabilize binding. Overall, these analyses suggest that the new candidates may achieve stable binding through balanced electrostatic complementarity, potentially contributing to their predicted affinity for PAK4 and NAMPT.

Interestingly, among the top 50 candidates, the kinase landscape showed a predominance of tyrosine kinase inhibitors, with a smaller subset associated with serine/threonine kinase-related signaling. Although PAK4 is a serine/threonine kinase and NAMPT is not a kinase, both represent key oncogenic regulators involved in complementary signaling and metabolic pathways. In addition, most tyrosine kinase inhibitors may indirectly modulate downstream serine/threonine kinase pathways through signaling cross-talk, particularly within PI3K–AKT–mTOR and stress-response networks [5,65,66,67]. To further interpret these findings at the pathway level, compounds were categorized based on their known kinase or signaling activities. First, a group of clinically approved receptor tyrosine kinase inhibitors, including Afatinib, Osimertinib, Tucatinib, and Lazertinib, primarily targeting EGFR or HER2 in non-small-cell lung cancer and HER2-positive breast cancer, converge on downstream PI3K–AKT–mTOR and MAPK signaling cascades, which regulate cellular proliferation and survival [68,69,70]. Second, compounds targeting central growth and stress response pathways, including Temsirolimus (mTOR inhibitor approved for renal cell carcinoma) [71], Gedatolisib (dual PI3K/mTOR inhibitor investigated in breast and endometrial cancers) [66,71], and Adavosertib (WEE1 checkpoint kinase inhibitor used in ovarian and solid tumors) [72], modulate nutrient sensing, translational control, and replication stress, reflecting coordinated control of cellular homeostasis [73,74,75,76,77]. Third, multi-target or broader spectrum kinase inhibitors, including Ponatinib (BCR–ABL and multi receptor tyrosine kinase inhibitor approved for chronic myeloid leukemia and Ph+ acute lymphoblastic leukemia), Avapritinib (KIT and PDGFRA inhibitor used in gastrointestinal stromal tumors), and UM-164 (SRC and p38 MAPK modulator), exhibit higher signaling network promiscuity [78,79,80]. Notably, UM-164 links more directly to MAPK-related serine/threonine signaling, suggesting partial overlap with stress-response kinase networks [80,81,82,83,84]. Collectively, these compounds converge on PI3K–AKT–mTOR, MAPK, and cell cycle regulatory pathways, reflecting a shared functional architecture across cellular growth and stress adaptation, with partial alignment to serine/threonine kinase–associated signaling axes [15,19,25,75]. Meanwhile, the predicted dual PAK4 and NAMPT inhibition may arise from binding site level promiscuity, where kinase inhibitors interact with the ATP-binding pocket of PAK4, while also potentially engaging the nicotinamide (NAM) binding site of NAMPT. Although these proteins belong to distinct functional classes, convergence at the level of ligand binding pockets may allow structurally flexible compounds to interact with both targets, supporting the possibility of dual-target activity. Importantly, several of the identified candidates are already FDA approved oncology drugs, including Afatinib, Osimertinib, Tucatinib, Temsirolimus, Ponatinib, and Avapritinib [35,73,78,85]. These clinically validated agents highlight a direct drug repurposing opportunity, as their established safety, pharmacokinetics, and dosing profiles enable faster translational evaluation than de novo development [86,87,88,89]. Even compounds not yet FDA approved but in advanced clinical development, such as Gedatolisib and Adavosertib, further support the clinical relevance of these findings [71,77]. Collectively, these drugs can potentially be used for cancers with high levels of PAK4 or NAMPT, even if that was not their original purpose. Future work could extend screening beyond traditionally cancer-associated agents to include structurally and pharmacologically diverse compounds, which may uncover additional candidates with unanticipated activity in oncogenic signaling and metabolic pathways.

Prediction of pharmacokinetic properties indicated that most selected compounds fell within acceptable ranges for key parameters, including molecular weight (approximately 480–650 g/mol), lipophilicity (consensus LogP values spanning 2.22 to 5.86), and topological polar surface area (TPSA; 88.61–241.96 Å^2^). These ranges are consistent with commonly used criteria for oral drug-likeness and membrane permeability, where molecular weight around or below ~500–600 g/mol and moderate TPSA (<140 Å^2^, with optimal permeability often <90 Å^2^) are generally associated with improved gastrointestinal absorption [90]. Lipophilicity also contributes to this balance, as moderate LogP values support both membrane permeation and aqueous solubility. Overall, the observed distributions suggest that most compounds possess physicochemical properties compatible with favorable oral bioavailability [91]. Many small-molecule compounds, including PAK4 and NAMPT-targeting compounds, are limited by low bioavailability and suboptimal pharmacokinetic properties [1,46,61,92,93]. High predicted gastrointestinal absorption for many candidates suggests the possibility of oral administration. In addition, limited predicted blood–brain barrier penetration for most compounds may reduce central nervous system-related adverse effects, which is desirable for many therapeutic applications. Compared with earlier inhibitors, several candidates showed improved solubility and more favorable cytochrome P450 interaction profiles, suggesting potentially improved pharmacokinetic properties and a lower predicted risk of drug–drug interactions. However, these predictions require experimental testing, as CYP inhibition and drug–drug interaction potential cannot be definitively established from computational predictions alone. These results suggest that the newly identified compounds may possess improved pharmacokinetic characteristics suitable for further development. Toxicity prediction also supported the potential advantages of the newly identified compounds. Most candidates showed low predicted risk for mutagenicity and cardiotoxicity compared with KPT-9274. Several compounds also demonstrated lower predicted hepatotoxicity, nephrotoxicity and cardiotoxicity. Several PAK4 and NAMPT inhibitors, including KPT-9274, PF-3758309, APO866 (FK866), CHS-828, and OT-82, have shown systemic toxicities such as anemia, thrombocytopenia, gastrointestinal effects, and off-target or pharmacokinetic limitations in preclinical and clinical studies [10,31,44,46,94]. Pan-group I PAK inhibitors (PAK1–3), which share structural similarities with PAK4 of Group II, have also shown acute cardiovascular toxicities in clinical studies [10,28]. Although in silico toxicity prediction cannot replace experimental testing, these results suggest that expanding the structural diversity of dual inhibitors may help identify safer molecules. Careful experimental validation will be required to confirm these predictions.

In addition to molecular docking studies, several compounds were also evaluated in biochemical assays. These compounds were selected from the top 50 candidates identified through the computational screening of 1173 inhibitors. These candidates represented diverse kinase inhibitor classes and target profiles, including inhibitors of serine/threonine kinases and tyrosine kinases. This diversity provided an opportunity to investigate whether compounds with distinct kinase-targeting profiles could also exhibit inhibitory activity against PAK4, a serine/threonine kinase, and NAMPT. The NAMPT activity assay demonstrated that several of the top-ranked compounds inhibited NAMPT enzymatic activity, although their inhibitory potency varied substantially. At 100 μM, CGP77675 and Copanlisib reduced NAMPT activity to approximately 5–10%, indicating relatively strong inhibition under the assay conditions. Tucatinib and YKL-5-124 also showed NAMPT inhibitory activity, reducing NAMPT activity to 56.55% and 45.22%, respectively, whereas NG25 exhibited weaker inhibition, with 57.44% NAMPT activity. In comparison, FK866, a well-established NAMPT inhibitor used as a positive control, reduced NAMPT activity to 4.68% at 100 μM. The concentration-dependent analysis further indicated that CGP77675 and Copanlisib displayed stronger NAMPT inhibitory activity than the other tested candidates, whereas YKL-5-124 and NG25 showed weaker inhibition. Dose–response analysis further quantified the NAMPT inhibitory activity of the tested compounds, with Copanlisib, Tucatinib, and CGP77675 exhibiting IC_50_ values of 5.8, 11.4, and 13.8 μM, respectively, while YKL-5-124 and NG25 showed weaker potency.

The PAK4 kinase assays demonstrated clear concentration-dependent inhibition by several of the selected compounds. Staurosporine and PF-3758309, used as positive controls, showed strong inhibition of PAK4 activity across the tested concentration range. Phloretin, a naturally occurring flavonoid, showed little inhibition and served as a negative control. Among the selected candidates, CGP77675, Copanlisib, YKL-5-124, and Tucatinib exhibited concentration-dependent inhibition of PAK4 activity, with IC_50_ values of 4.79, 5.02, 4.10, and 5.86 μM, respectively. These results indicate that several compounds selected from the top 50 computational candidates can directly inhibit recombinant PAK4 kinase activity. In contrast, KPT-9274 did not show strong inhibition in the ATP-dependent PAK4 kinase assay. This finding is consistent with its mechanism of action, as KPT-9274 is an allosteric PAK4 inhibitor rather than an ATP-competitive inhibitor. Therefore, its inhibitory activity may not be adequately reflected in an assay that evaluates ATP-dependent catalytic activity of recombinant PAK4. The lack of strong inhibition observed for KPT-9274 in this assay should therefore not be interpreted as evidence that it lacks PAK4 inhibitory activity. Instead, these results highlight the importance of considering the mechanism of inhibition when selecting an appropriate biochemical assay. Overall, the concentration-dependent inhibition observed for CGP77675, Copanlisib, YKL-5-124, and Tucatinib, together with their NAMPT inhibitory activity, supports their potential as dual PAK4/NAMPT inhibitor candidates and warrants further investigation in cellular and mechanistic studies.

Several compounds demonstrated dual PAK4/NAMPT inhibitory activity. Tucatinib, CGP77675, Copanlisib, and YKL-5-124 showed inhibition of both enzymes, although the extent of inhibition varied among compounds. Tucatinib, a HER2-directed tyrosine kinase inhibitor, showed substantial inhibition of both PAK4 and NAMPT. CGP77675 and Copanlisib also demonstrated strong inhibition of both enzymes, whereas YKL-5-124 showed stronger PAK4 inhibition but weaker NAMPT inhibitory activity.

The findings from the enzymatic assays as well as the molecular docking studies illustrate the potential polypharmacological nature of the identified compounds. Such multi-target activity may provide therapeutic advantages by simultaneously affecting complementary signaling and metabolic pathways, but it may also complicate the attribution of cellular effects to a specific target and contribute to potential off-target effects. Furthermore, the current biochemical assays do not indicate whether the biological effects of these compounds would be primarily mediated by PAK4, NAMPT, their established targets, or other potential off-target mechanisms. It is also important to note that we cannot rule out the possibility that some of the effects of these compounds on PAK4 and NAMPT may be due to nonspecific effects, and further studies will be needed to assess this. Further cellular, genetic, and broader target-profiling studies will therefore be required to determine the relative contributions of PAK4 and NAMPT to their biological activities and to further evaluate their selectivity and therapeutic potential.

Overall, this study shows that computational screening can successfully identify novel compounds with predicted dual inhibition of PAK4 and NAMPT, favorable pharmacokinetics and safety profiles. The combination of docking, ADME analysis, and toxicity prediction provides a mechanism for prioritizing candidates before experimental testing. The top-ranked compounds identified in this work represent promising starting points for further biochemical assays, cell-based studies, and structure optimization. Future studies should focus on further experimental validation of target inhibition, evaluation of cellular activity, and structural optimization to improve their effectiveness. These efforts may contribute to the development of improved dual inhibitors with enhanced therapeutic potential.

## 4. Materials and Methods

### 4.1. Molecular Docking Analysis

A targeted virtual screening workflow was established to identify potential dual NAMPT/PAK4 inhibitors. The crystal structures of NAMPT (PDB ID: 5U2N) and PAK4 (PDB ID: 4FIE) were retrieved from the Protein Data Bank and processed using MOE 2022 (Molecular Operating Environment, 2022 version, accessed on 30 August 2026). A curated dataset of 1173 inhibitors with reported anticancer activity was downloaded from the PubChem database (https://pubchem.ncbi.nlm.nih.gov/, accessed on 28 December 2025) and constituted the virtual screening library. Previously characterized compounds, including KPT-9274, GNE2861, LCH7749944, and PF-3758309, were not included in the virtual screening library because they had been investigated in our previous study [38]. Their docking scores were used as reference values for candidate selection. Compounds with predicted binding affinities comparable to KPT-9274 for NAMPT (−7.25 kcal/mol) and strong PAK4 inhibitors (≤−7.00 kcal/mol) were selected, resulting in 48 compounds, including KPT-9274, for further ADME and toxicity analyses. Following receptor preparation, molecular docking simulations were performed within the catalytic binding pockets of both proteins. Docking poses were evaluated based on binding energy scores and interaction patterns. Compounds demonstrating strong predicted affinity for both targets were prioritized as putative dual inhibitors. Detailed ligand–receptor interactions, including hydrogen bonds, hydrophobic contacts, and π-related interactions, were visualized and analyzed using the MOE graphical interface.

### 4.2. ADME Analysis

The ADME properties of all candidate compounds were assessed using the SwissADME web server (http://www.swissadme.ch/, accessed on 12 January 2026). Canonical SMILES obtained from the PubChem database were submitted for in silico evaluation. Key pharmacokinetic parameters, including gastrointestinal (GI) absorption, blood–brain barrier (BBB) permeability, P-glycoprotein (P-gp) substrate prediction, and cytochrome P450 (CYP) inhibition profiles, were analyzed. Drug-likeness was further evaluated based on Lipinski’s rule of five and additional medicinal chemistry filters (Veber, Ghose, Egan, and Muegge). In addition, molecular descriptors such as molecular weight, lipophilicity (logP), hydrogen bond donors and acceptors, topological polar surface area (TPSA), and bioavailability score were recorded to prioritize compounds with favorable pharmacokinetic characteristics for subsequent biological studies.

### 4.3. Toxicity Analysis

A total of 48 top-ranked candidates were selected for in silico oral toxicity evaluation using the ProTox 3.0 platform (https://tox.charite.de/protox3/, accessed on 30 January 2026). Canonical SMILES of all compounds were retrieved from the PubChem database and submitted for analysis. The predicted oral toxicity class (1–6) was used to estimate acute oral toxicity, where class 1 represents the highest toxicity and class 6 indicates the lowest toxicity.

### 4.4. Molecular Electrostatic Potential (MEP) Analysis

The MEP maps were generated using the Molecular Operating Environment (MOE, 2022 version, accessed on 30 August 2026). The 3D structures of selected compounds were first energy-minimized to obtain stable conformations for surface analysis. Electrostatic potential calculations were performed using the implemented force field within MOE and subsequently mapped onto the solvent-accessible molecular surface. The MEP surfaces were visualized and analyzed in MOE to characterize charge distribution features, including electron-rich and electron-deficient regions, and to identify potential electrostatic interaction sites relevant to ligand–target recognition.

### 4.5. NAMPT Enzymatic Activity Assay

The effects of top candidate drugs on NAMPT enzymatic activity were evaluated using a recombinant NAMPT cell-free assay with a CycLex NAMPT Colorimetric Assay Kit (Cat. No. CY-1251; CycLex Co., Ltd., Nagano, Japan), following the manufacturer’s protocol. Briefly, a two-step enzyme-coupled assay was performed to quantify NAMPT activity. Two-Step Assay Mixture I and Two-Step Assay Mixture II were freshly prepared within 30 min before use. Assay Mixture I contained NAMPT assay buffer, ATP, nicotinamide phosphoribosyltransferase substrate components including nicotinamide and phosphoribosyl pyrophosphate (PRPP), and nicotinamide mononucleotide adenylyltransferase 1 (NMNAT1). Assay Mixture II contained water-soluble tetrazolium salt (WST-1), alcohol dehydrogenase (ADH), diaphorase, and ethanol.

Recombinant NAMPT was incubated with the indicated top candidate drugs or control compounds in the presence of Assay Mixture I at 30 °C for 60 min. Subsequently, Assay Mixture II was added to each reaction and incubated at 30 °C for an additional 30 min. Absorbance was measured at 450 nm immediately after the addition of Assay Mixture II (0 min) and after 30 min of incubation using an Infinite 200 PRO microplate reader (Tecan Austria GmbH, Grödig, Austria). NAMPT activity was calculated from the change in absorbance at 450 nm relative to the vehicle-treated control.

### 4.6. PAK4 Enzymatic Activity Assay

PAK4 kinase activity was determined using the ADP-Glo™ + PAK4 Kinase Enzyme System (Promega, Madison, WI, USA; Cat. No. V9451) according to the manufacturer’s instructions. This luminescence-based assay measures ADP production during the kinase reaction as an indicator of PAK4 activity. Briefly, test compounds or vehicle control (DMSO) were added to a 384-well low-volume plate containing recombinant human PAK4 kinase, kinase substrate, and ATP in PAK4 kinase buffer (40 mM Tris-HCl, pH 7.5, 20 mM MgCl_2_, 0.1 mg/mL bovine serum albumin, and 50 μM dithiothreitol). The kinase reaction was incubated at room temperature for 60 min, after which ADP-Glo™ Reagent was added to terminate the reaction and deplete the remaining ATP. Following a 40 min incubation at room temperature, Kinase Detection Reagent was added to convert ADP to ATP and generate a luminescent signal through a luciferase/luciferin reaction. After incubation for 30 min at room temperature, luminescence was measured using an Infinite 200 PRO microplate reader (Tecan Austria GmbH, Grödig, Austria). PAK4 activity was calculated relative to vehicle-treated controls, and percent inhibition was determined for each compound. For IC_50_ analysis, compounds were tested over a range of concentrations, and dose–response curves were fitted using a four-parameter logistic model in OriginPro 2023 (OriginLab Corporation, Northampton, MA, USA). IC_50_ values were calculated from the fitted curves.

### 4.7. Statistical Analysis

All data were presented as mean ± standard deviation (SD), shown as error bars. An independent Student’s *t*-test was used to compare the means of the two groups. Differences were considered statistically significant at *p* < 0.05. All the statistical analyses were conducted using SPSS software (Version 17.0, SPSS, Inc., Chicago, IL, USA).

## 5. Conclusions

In conclusion, this study used an integrated computational strategy combining virtual screening and molecular docking. The results were used for ADME prediction, toxicity estimation, and molecular electrostatic potential analysis to identify potential dual inhibitors targeting both PAK4 and NAMPT. Several top-ranked compounds showed strong predicted binding affinities for both targets, forming stable interactions within the ATP-binding pocket of PAK4 and the nicotinamide-binding site of NAMPT. The selected candidates also exhibited favorable electrostatic complementarity and some properties favorable relative to KPT-9274, although limitations in gastrointestinal absorption, CYP interactions, and predicted toxicity were observed for several compounds. Importantly, biochemical assays provided experimental support for PAK4 and NAMPT inhibitory activity among several selected compounds, including CGP77675, Copanlisib, Tucatinib, YKL-5-124, and NG25, although their inhibitory potencies varied. These findings expand the chemical diversity of potential dual PAK4 and NAMPT inhibitors and suggest that the top-ranked compounds warrant further validation through cell-based studies, genetic target validation, and structure optimization.

## Figures and Tables

**Table 1:** Ligand interaction report of the top candidate compounds docked to the PAK4 protein. Specific interactions between ligand functional groups or aromatic rings and PAK4 amino acid residues are shown, including hydrogen bonding, π–hydrogen, and π–cation interactions. Lower docking scores indicate stronger binding affinities between the protein and the compounds. Shorter interaction distances together with more negative interaction energies generally indicate stronger and more favorable ligand–PAK4 interactions. The top candidate compounds exhibited strong predicted binding affinities to PAK4 relative to KPT-9274. Based on their predicted NAMPT scores (see Table 2), the top 10 compounds were selected for their high predicted activity against both PAK4 and NAMPT, highlighting their potential as dual inhibitors. KPT-9274 serves as the control in the first row of the table.

No.	Drugs [a]	PAK4 Score (kcal/mol) [b]	Ligand	Receptor		Interaction	Distance	E (kcal/mol)
1	KPT-9274	−7.5	N10	OG	SER466 (A) [c]	H-donor	2.97	−1.6
			6-ring	CG	GLN434 (A)	π-H	3.97	−0.8
2	Afatinib	−7.18	N8	O	LEU398 (B)	H-donor	3.34	−0.7
			6-ring	N	ALA402 (B)	π-H	3.90	−2.1
3	Tucatinib	−7.89	5-ring	NZ	LYS350 (B)	π-cation	4.01	−1.4
			6-ring	N	ALA402 (B)	π-H	3.90	−1.3
4	Lazertinib	−7.15	6-ring	CD	LYS51 (B)	π-H	3.77	−0.5
5	Osimertinib	−7.01	N8	NH1	ARG586 (B)	H-acceptor	3.39	−2.9
6	JTE-952	−7.43	O5	OE1	GLU361 (A)	H-donor	2.79	−2.0
			O2	N	ARG360 (A)	H-acceptor	2.99	−1.3
			N8	NH2	ARG360 (A)	H-acceptor	3.12	−0.8
7	Temsirolimus	−7.50	O7	NZ	LYS351 (B)	H-acceptor	3.11	−0.9
			O10	NZ	LYS351 (B)	H-acceptor	2.86	−2.6
8	Avapritinib	−7.15	5-ring	NH2	ARG453 (B)	π-cation	4.07	−1.4
			6-ring	NH2	ARG453 (B)	π-cation	4.60	−1.5
9	UM-164	−7.56	6-ring	CD2	LEU567 (A)	π-H	4.15	−0.6
10	ONO-7579	−8.41	F3	N	LEU475 (A)	H-acceptor	2.96	−0.7
			F5	NH2	ARG489 (A)	H-acceptor	3.16	−0.7
			O6	CE	LYS473 (A)	H-acceptor	3.47	−0.5
			6-ring	CA	SER300 (A)	π-H	4.11	−0.5
			6-ring	CA	LYS345 (B)	π-H	4.24	−0.7

[a] The inhibitors used in the study. [b] Docking Score between inhibitors and PAK4. [c] A lower docking score indicates stronger predicted binding affinities between the protein and the compound. (A) and (B) indicate chains A and B of the receptor, respectively. The crystal structures of NAMPT (PDB ID: 5U2N) and PAK4 (PDB ID: 4FIE) were retrieved from the Protein Data Bank.

**Table 2:** Ligand interaction report of the top candidate compounds docked to the NAMPT protein. Specific interactions between ligand functional groups or aromatic rings and NAMPT amino acid residues are shown, including hydrogen bonding, π–hydrogen, and π–cation interactions. Lower docking scores indicate stronger binding affinities between NAMPT and the compounds. Shorter interaction distances, together with more negative interaction energies, generally reflect stronger and more favorable ligand–NAMPT interactions. The top candidate compounds also exhibited strong predicted binding affinities to PAK4 relative to KPT-9274. Considering their predicted PAK4 scores (see Table 1), the top 10 compounds were selected based on high predicted activity against both PAK4 and NAMPT, highlighting their potential as dual inhibitors. KPT-9274 serves as the control in the first row of the table.

No.	Drugs [a]	NAMPT Score (kcal/mol) [b]	Ligand	Receptor		Interaction	Distance	E (kcal/mol)
1	KPT-9274	−7.25	N8	O	ASP393 (B) [c]	H-donor	3.37	−0.5
			N9	NH1	ARG40 (B)	H-acceptor	3.22	−1.7
			N9	NH1	ARG40 (B)	H-acceptor	3.07	−3.4
			6-ring	CE	LYS423 (B)	π-H	3.72	−0.5
2	Afatinib	−8.67	6-ring	NH2	ARG392 (A)	π-cation	3.249	−1.0
			6-ring	CA	ASP354 (B)	π-H	4.27	−0.6
			6-ring	N	GLY355 (B)	π-H	3.44	−0.5
3	Tucatinib	−8.66	6-ring	N	GLY383 (A)	π-H	4.08	−1.4
			6-ring	CA	GLY383 (A)	π-H	4.77	−0.8
			5-ring	6-ring	PHE193 (A)	π-π	3.67	−0.0
4	Lazertinib	−8.34	6-ring	CA	PHE9 (A)	π-H	4.48	−0.5
			6-ring	N	ASN10 (A)	π-H	4.37	−0.7
5	Osimertinib	−8.32	N7	O	PHE193 (A)	H-donor	2.96	−4.1
			N4	NZ	LYS389 (A)	H-acceptor	3.41	−5.1
			5-ring	CB	PHE193 (A)	π-H	4.56	−0.7
			6-ring	NZ	LYS400 (B)	π-cation	4.59	−1.4
6	JTE-952	−8.15	O6	OE1	GLN201 (A)	H-donor	2.99	−1.5
			O2	CE	LYS68 (A)	H-acceptor	3.00	−1.0
7	Temsirolimus	−7.93	O4	NH2	ARG434 (A)	H-acceptor	3.45	−0.9
			O9	NH1	ARG434 (A)	H-acceptor	3.00	−2.8
8	Avapritinib	−7.90	N5	CE	LYS68 (B)	H-acceptor	3.16	−0.8
			N8	NZ	LYS68 (A)	H-acceptor	3.63	−1.8
9	UM-164	−7.85	S1	O	THR304 (A)	H-donor	3.75	−1.6
			N12	O	GLN305 (A)	H-donor	3.09	−2.1
			O7	NH2	ARG349 (A)	H-acceptor	2.95	−5.5
			6-ring	NE	ARG349 (A)	π-cation	4.06	−0.9
10	ONO-7579	−7.85	O6	NZ	LYS68 (B)	H-acceptor	3.40	−1.4
			6-ring	CB	GLU202 (A)	π-H	4.23	−0.9

[a] The inhibitors used in the study. [b] Docking Score between inhibitors and NAMPT. [c] A lower docking score indicates stronger binding affinities between a protein and a drug. (A) and (B) indicate chains A and B of the receptor, respectively. The crystal structures of NAMPT (PDB ID: 5U2N) and PAK4 (PDB ID: 4FIE) were retrieved from the Protein Data Bank.

**Table 3:** Predicted ADME properties of top candidate compounds. The table summarizes physicochemical and pharmacokinetic properties, including molecular weight (M.W.), hydrogen bond donors and acceptors, lipophilicity (Log P), water solubility, gastrointestinal (GI) absorption, blood–brain barrier (BBB) permeability, P-glycoprotein (P-gp) substrate status, and cytochrome P450 (CYP) enzyme inhibition profiles. These properties provide an initial assessment of drug-likeness and potential pharmacokinetic behavior for the top candidate compounds.

Compounds Name	KPT-9274	Afatinib	Tucatinib	Lazertinib	Osimertinib	JTE-952	Temsirolimus	Avapritinib	UM-164	ONO-7579
Physicochemical Properties										
M.W. (g/mol)	610.62	485.94	480.52	554.64	485.58	518.6	1030.29	498.56	640.68	578.95
No. of heavy atoms	45	34	36	41	36	38	73	37	45	39
No. of aromatic heavy atoms	27	16	25	23	21	18	0	26	23	24
Fraction Csp3	0.17	0.29	0.19	0.27	0.22	0.4	0.75	0.27	0.3	0.08
No. of rotatable bonds	9	9	6	11	11	13	11	5	12	9
No. of H-bond acceptors	7	7	7	7	5	7	16	7	10	10
No. of H-bond Donors	2	2	2	2	3	2	4	1	4	3
Molar Refractivity	170.7	129.9	141.66	162.47	145.53	145.96	279.9	144.37	172.84	138.01
TPSA (Å^2^)	101.46	88.61	110.85	109.67	98.41	101.35	241.96	106.29	163.85	157.57
Lipophilicity										
Log *P_o/w_* (iLOGP)	4.22	4.27	4.14	4.34	3.27	4.51	6.82	3.54	3.6	3.22
Log *P_o/w_* (XLOGP3)	6.11	3.64	3.99	3.37	3.77	2.86	5.55	1.86	4.68	4.14
Log *P_o/w_* (WLOGP)	7.71	4.62	4.52	3.38	4.31	2.91	5.34	2.16	4.94	7.49
Log *P_o/w_* (MLOGP)	4.06	2.3	3.01	1.75	1.51	1.48	0.3	2.37	1.9	3.07
Log *P_o/w_* (SILICOS-IT)	7.18	3.82	3.19	2.7	3.45	4.9	3.98	1.18	4.53	3.2
Consensus Log *P_o/w_*	5.86	3.73	3.77	3.11	3.26	3.33	4.4	2.22	3.93	4.23
Water Solubility										
Log *S* (ESOL)	−7.33	−4.9	−5.45	−5.09	−4.93	−4.35	−9	−4.29	−6.35	−5.9
Solubility (mg/mL)	2.89 × 10^−5^	6.11 × 10^−3^	1.70 × 10^−3^	4.50 × 10^−3^	5.69 × 10^−3^	2.32 × 10^−2^	1.03 × 10^−6^	2.54 × 10^−2^	2.88 × 10^−4^	7.30 × 10^−4^
Class	Poorly soluble	Moderately soluble	Moderately soluble	Moderately soluble	Moderately soluble	Moderately soluble	Poorly soluble	Moderately soluble	Poorly soluble	Moderately soluble
Log *S* (Ali)	−8.02	−5.19	−6.02	−5.35	−5.53	−4.65	−10.39	−3.71	−7.85	−7.16
Solubility (mg/mL)	5.80 × 10^−6^	3.14 × 10^−3^	4.59 × 10^−4^	2.47 × 10^−3^	1.43 × 10^−3^	1.17 × 10^−2^	4.18 × 10^−8^	9.65 × 10^−2^	9.08 × 10^−6^	4.04 × 10^−5^
Class	Poorly soluble	Moderately soluble	Poorly soluble	Moderately soluble	Moderately soluble	Moderately soluble	Insoluble	Soluble	Poorly soluble	Poorly soluble
Log *S* (SILIOS-IT)	−11.78	−7.59	−9.12	−8.3	−8.68	−7.47	−5.74	−6.88	−9.47	−9.81
Solubility (mg/mL)	1.00 × 10^−9^	1.23 × 10^−5^	3.65 × 10^−7^	2.78 × 10^−6^	1.01 × 10^−6^	1.76 × 10^−5^	1.89 × 10^−3^	6.56 × 10^−5^	2.16 × 10^−7^	9.05 × 10^−8^
Class	Insoluble	Poorly soluble	Poorly soluble	Poorly soluble	Poorly soluble	Poorly soluble	Moderately soluble	Poorly soluble	Poorly soluble	Poorly soluble
Pharmacokinetics										
GI absorption	Low	High	High	High	High	High	Low	High	Low	Low
BBB permeant	No	No	No	No	No	No	No	No	No	No
P-gp substrate	No	Yes	Yes	Yes	Yes	Yes	Yes	Yes	No	No
CYP1A2 inhibitor	No	No	Yes	No	Yes	No	No	No	No	Yes
CYP2C19 inhibitor	No	Yes	Yes	Yes	Yes	Yes	No	No	Yes	Yes
CYP2C9 inhibitor	No	Yes	Yes	Yes	Yes	Yes	No	Yes	Yes	Yes
CYP2D6 inhibitor	Yes	Yes	Yes	Yes	Yes	Yes	No	Yes	No	No
CYP3A4 inhibitor	No	Yes	Yes	Yes	Yes	Yes	No	Yes	Yes	Yes
Log K_p_ (skin permeation)	−5.69	−6.68	−6.4	−7.29	−6.59	−7.43	−8.64	−8.02	−6.89	−6.89

**Table 4:** Predicted toxicity properties of top candidate compounds. The table summarizes in silico toxicity predictions, including oral toxicity class (1–6, where higher values indicate lower acute toxicity), organ toxicity, toxicity endpoints, nuclear receptor signaling pathways, molecular initiating events, and cytochrome P450-related metabolic interactions. “Active” indicates that a compound is predicted to exhibit a potential toxic effect or interaction for the specified endpoint, whereas “inactive” indicates no or low predicted toxicity or interaction.

Compounds Name	KPT-9274	Afatinib	Tucatinib	Lazertinib	Osimertinib	JTE-952	Temsirolimus	Avapritinib	UM-164	ONO-7579
Predicted oral toxicity class (1–6 good)	4	4	5	4	3	4	5	5	5	4
Organ toxicity										
Hepatotoxicity	Inactive	Active	Active	Inactive	Inactive	Inactive	Inactive	Inactive	Active	Active
Neurotoxicity	Active	Active	Active	Active	Active	Active	Active	Active	Active	Active
Nephrotoxicity	Inactive	Inactive	Inactive	Inactive	Inactive	Active	Inactive	Active	Inactive	Inactive
Respiratory toxicity	Active	Active	Active	Active	Active	Active	Active	Active	Active	Active
Cardiotoxicity	Inactive	Inactive	Inactive	Inactive	Inactive	Inactive	Inactive	Inactive	Inactive	Inactive
Toxicity endpoints										
Carcinogenicity	Active	Active	Active	Active	Active	Active	Active	Active	Active	Active
Immunotoxicity	Inactive	Inactive	Inactive	Inactive	Inactive	Inactive	Inactive	Inactive	Inactive	Inactive
Mutagenicity	Inactive	Inactive	Inactive	Inactive	Inactive	Inactive	Inactive	Inactive	Inactive	Inactive
Cytotoxicity	Active	Active	Active	Active	Active	Inactive	Active	Inactive	Active	Active
Blood–Brain Barrier (BBB)	Inactive	Active	Active	Inactive	Active	Inactive	Active	Inactive	Active	Active
Ecotoxicity	Active	Active	Active	Active	Active	Active	Active	Active	Active	Active
Clinical toxicity	Inactive	Inactive	Inactive	Inactive	Inactive	Active	Inactive	Active	Inactive	Inactive
Nutritional toxicity	Inactive	Inactive	Inactive	Inactive	Inactive	Inactive	Inactive	Inactive	Inactive	Inactive
Tox21-nuclear receptor signaling pathways										
Aryl hydrocarbon Receptor (AhR)	Inactive	Inactive	Inactive	Inactive	Inactive	Inactive	Inactive	Inactive	Inactive	Inactive
Androgen Receptor (AR)	Inactive	Inactive	Inactive	Inactive	Inactive	Active	Inactive	Active	Inactive	Inactive
Androgen Receptor Ligand Binding Domain (AR-LBD)	Inactive	Inactive	Inactive	Inactive	Inactive	Inactive	Inactive	Inactive	Inactive	Inactive
Aromatase	Inactive	Inactive	Inactive	Inactive	Inactive	Inactive	Inactive	Inactive	Inactive	Inactive
Estrogen Receptor Alpha (ER)	Inactive	Inactive	Inactive	Inactive	Inactive	Inactive	Inactive	Inactive	Inactive	Inactive
Estrogen Receptor Ligand Binding Domain (ER-LBD)	Inactive	Inactive	Inactive	Inactive	Inactive	Inactive	Inactive	Inactive	Inactive	Inactive
Peroxisome Proliferator Activated Receptor Gamma (PPARγ)	Inactive	Inactive	Inactive	Inactive	Inactive	Inactive	Inactive	Inactive	Inactive	Inactive
Nuclear factor (erythroid-derived 2)-like 2/antioxidant responsive element (Nrf2/ARE)	Inactive	Inactive	Inactive	Inactive	Inactive	Active	Inactive	Active	Inactive	Inactive
Heat shock factor response element (HSE)	Inactive	Inactive	Inactive	Inactive	Inactive	Inactive	Inactive	Inactive	Inactive	Inactive
Mitochondrial Membrane Potential (MMP)	Inactive	Inactive	Inactive	Inactive	Inactive	Inactive	Inactive	Inactive	Inactive	Inactive
Phosphoprotein (Tumor Suppressor) p53	Inactive	Inactive	Inactive	Inactive	Inactive	Inactive	Inactive	Inactive	Inactive	Inactive
ATPase family AAA domain-containing protein 5 (ATAD5)	Inactive	Inactive	Inactive	Inactive	Inactive	Inactive	Inactive	Inactive	Inactive	Inactive
Molecular initiating events										
Thyroid hormone receptor alpha (THRα)	Inactive	Inactive	Inactive	Active	Inactive	Inactive	Inactive	Inactive	Inactive	Inactive
Thyroid hormone receptor beta (THRβ)	Inactive	Inactive	Inactive	Inactive	Inactive	Inactive	Inactive	Inactive	Inactive	Inactive
Transthyretin (TTR)	Inactive	Inactive	Inactive	Active	Inactive	Inactive	Inactive	Inactive	Inactive	Inactive
Ryanodine receptor (RYR)	Inactive	Inactive	Inactive	Inactive	Inactive	Inactive	Inactive	Inactive	Inactive	Inactive
GABA receptor (GABAR)	Inactive	Inactive	Inactive	Active	Inactive	Inactive	Inactive	Inactive	Inactive	Inactive
Glutamate N-methyl-D-aspartate receptor (NMDAR)	Inactive	Active	Active	Active	Inactive	Inactive	Inactive	Inactive	Active	Active
alpha-amino-3-hydroxy-5-methyl-4-isoxazolepropionate receptor (AMPAR)	Inactive	Inactive	Inactive	Inactive	Inactive	Inactive	Inactive	Inactive	Inactive	Inactive
Kainate receptor (KAR)	Inactive	Inactive	Inactive	Inactive	Inactive	Active	Inactive	Active	Inactive	Inactive
Acetylcholinesterase (AChE)	Inactive	Inactive	Inactive	Active	Inactive	Inactive	Inactive	Inactive	Inactive	Inactive
Constitutive androstane receptor (CAR)	Active	Inactive	Inactive	Inactive	Inactive	Inactive	Inactive	Inactive	Inactive	Inactive
Pregnane X receptor (PXR)	Inactive	Inactive	Inactive	Active	Inactive	Inactive	Inactive	Inactive	Inactive	Inactive
NADH-quinone oxidoreductase (NADHOX)	Inactive	Inactive	Inactive	Inactive	Inactive	Inactive	Inactive	Inactive	Inactive	Inactive
Voltage gated sodium channel (VGSC)	Inactive	Inactive	Inactive	Inactive	Inactive	Inactive	Inactive	Inactive	Inactive	Inactive
Na+/I− symporter (NIS)	Inactive	Inactive	Inactive	Inactive	Inactive	Inactive	Inactive	Inactive	Inactive	Inactive

## Data Availability

The original contributions presented in this study are included in the article/Supplementary Materials. Further inquiries can be directed to the corresponding author.

## Supplementary Materials

The following supporting information can be downloaded at: https://www.mdpi.com/article/10.3390/ijms27177706/s1.

## References

[1] Wang Y., Minden A. (2021). The Use of Nanomedicine to Target Signaling by the PAK Kinases for Disease Treatment. Cells.

[2] Rane C.K., Minden A. (2019). P21 activated kinase signaling in cancer. Semin. Cancer Biol..

[3] Kumar R., Sanawar R., Li X., Li F. (2017). Structure, biochemistry, and biology of PAK kinases. Gene.

[4] Shahinozzaman M., Ishii T., Ahmed S., Halim M.A., Tawata S. (2020). A computational approach to explore and identify potential herbal inhibitors for the p21-activated kinase 1 (PAK1). J. Biomol. Struct. Dyn..

[5] Liu H., Liu K., Dong Z. (2021). The Role of p21-Activated Kinases in Cancer and Beyond: Where Are We Heading?. Front. Cell Dev. Biol..

[6] Binder P., Wang S., Radu M., Zin M., Collins L., Khan S., Li Y., Sekeres K., Humphreys N., Swanton E. (2019). Pak2 as a Novel Therapeutic Target for Cardioprotective Endoplasmic Reticulum Stress Response. Circ. Res..

[7] Lin H., Rothe K., Chen M., Wu A., Babaian A., Yen R., Zeng J., Ruschmann J., Petriv O.I., O’Neill K. (2020). The miR-185/PAK6 axis predicts therapy response and regulates survival of drug-resistant leukemic stem cells in CML. Blood.

[8] Han K., Zhou Y., Tseng K., Hu H., Li K., Wang Y., Gan Z., Lin S., Sun Y., Min D. (2018). PAK5 overexpression is associated with lung metastasis in osteosarcoma. Oncol. Lett..

[9] Wang J., Zhu Y., Chen J., Yang Y., Zhu L., Zhao J., Yang Y., Cai X., Hu C., Rosell R. (2020). Identification of a novel PAK1 inhibitor to treat pancreatic cancer. Acta Pharm. Sin. B.

[10] Li Y., Lu Q., Xie C., Yu Y., Zhang A. (2022). Recent advances on development of p21-activated kinase 4 inhibitors as anti-tumor agents. Front. Pharmacol..

[11] Yu X., Huang C., Liu J., Shi X., Li X. (2022). The significance of PAK4 in signaling and clinicopathology: A review. Open Life Sci..

[12] Najahi-Missaoui W., Quach N.D., Jenkins A., Dabke I., Somanath P.R., Cummings B.S. (2019). Effect of P21-activated kinase 1 (PAK-1) inhibition on cancer cell growth, migration, and invasion. Pharmacol. Res. Perspect..

[13] Grebeňová D., Holoubek A., Röselová P., Obr A., Brodská B., Kuželová K. (2019). PAK1, PAK1Delta15, and PAK2: Similarities, differences and mutual interactions. Sci. Rep..

[14] Mpilla G.B., Uddin H., Al-Hallak M.N., Aboukameel A., Li Y., Kim S.H., Beydoun R., Dyson G., Baloglu E., Senapedis W.T. (2021). PAK4-NAMPT Dual Inhibition Sensitizes Pancreatic Neuroendocrine Tumors to Everolimus. Mol. Cancer Ther..

[15] Yuan Y., Zhang H., Li D., Li Y., Lin F., Wang Y., Song H., Liu X., Li F., Zhang J. (2022). PAK4 in cancer development: Emerging player and therapeutic opportunities. Cancer Lett..

[16] Won S.-Y., Park J.-J., Shin E.-Y., Kim E.-G. (2019). PAK4 signaling in health and disease: Defining the PAK4-CREB axis. Exp. Mol. Med..

[17] Peytam F., Emamgholipour Z., Mousavi A., Moradi M., Foroumadi R., Firoozpour L., Divsalar F., Safavi M., Foroumadi A. (2023). Imidazopyridine-based kinase inhibitors as potential anticancer agents: A review. Bioorg. Chem..

[18] Belli S., Pesapane A., Servetto A., Esposito D., Napolitano F., Ascione C.M., Allotta A., Zambrano N., Marino F.Z., Franco R. (2023). Combined blockade of mTOR and p21-activated kinases pathways prevents tumour growth in KRAS-mutated colorectal cancer. Br. J. Cancer.

[19] Cheng F., Li M., Thorne R.F., Liu G., Zhang Y., Wu M., Liu L. (2022). P21-Activated Kinase 4 Pak4 Maintains Embryonic Stem Cell Pluripotency via Akt Activation. Stem Cells.

[20] Abril-Rodriguez G., Torrejon D.Y., Liu W., Zaretsky J.M., Nowicki T.S., Tsoi J., Puig-Saus C., Baselga-Carretero I., Medina E., Quist M.J. (2020). PAK4 inhibition improves PD-1 blockade immunotherapy. Nat. Cancer.

[21] Ashraf U., Ding Z., Deng S., Ye J., Cao S., Chen Z. (2021). Pathogenicity and virulence of Japanese encephalitis virus: Neuroinflammation and neuronal cell damage. Virulence.

[22] Pallesen L.T., Gustafsen C., Cramer J.F., Petersen S.V., Thirup S.S., Madsen P., Petersen C.M. (2020). PAK Kinases Target Sortilin and Modulate Its Sorting. Mol. Cell Biol..

[23] Gasparrini M., Audrito V. (2022). NAMPT: A critical driver and therapeutic target for cancer. Int. J. Biochem. Cell Biol..

[24] Yaku K., Okabe K., Hikosaka K., Nakagawa T. (2018). NAD Metabolism in Cancer Therapeutics. Front. Oncol..

[25] Guo C., Huang Q., Wang Y., Yao Y., Li J., Chen J., Wu M., Zhang Z., E M., Qi H. (2023). Therapeutic application of natural products: NAD(+) metabolism as potential target. Phytomedicine.

[26] Navas L.E., Carnero A. (2021). NAD(+) metabolism, stemness, the immune response, and cancer. Signal Transduct. Target. Ther..

[27] Amjad S., Nisar S., Bhat A.A., Shah A.R., Frenneaux M.P., Fakhro K., Haris M., Reddy R., Patay Z., Baur J. (2021). Role of NAD(+) in regulating cellular and metabolic signaling pathways. Mol. Metab..

[28] Wei Y., Xiang H., Zhang W. (2022). Review of various NAMPT inhibitors for the treatment of cancer. Front. Pharmacol..

[29] Hoxhaj G., Ben-Sahra I., Lockwood S.E., Timson R.C., Byles V., Henning G.T., Gao P., Selfors L.M., Asara J.M., Manning B.D. (2019). Direct stimulation of NADP+ synthesis through Akt-mediated phosphorylation of NAD kinase. Science.

[30] Ye C., Qi L., Li X., Wang J., Yu J., Zhou B., Guo C., Chen J., Zheng S. (2020). Targeting the NAD(+) salvage pathway suppresses APC mutation-driven colorectal cancer growth and Wnt/beta-catenin signaling via increasing Axin level. Cell Commun. Signal.

[31] Wen F., Gui G., Wang X., Ye L., Qin A., Zhou C., Zha X. (2024). Drug discovery targeting nicotinamide phosphoribosyltransferase (NAMPT): Updated progress and perspectives. Bioorg. Med. Chem..

[32] Garten A., Petzold S., Körner A., Imai S.-I., Kiess W. (2009). Nampt: Linking NAD biology, metabolism and cancer. Trends Endocrinol. Metab..

[33] Mylonakis A., Kozadinos A., Frountzas M., Kapetanakis E.I., Lidoriki I., Despotidis M., Karanikki E., Triantafyllou T., Theodorou D., Toutouzas K.G. (2025). The Role of Visfatin in Gastric and Esophageal Cancer: From Biomarker to Therapeutic Target. Cancers.

[34] Li N., Lopez M.A., Linares M., Kumar S., Oliva S., Martinez-Lopez J., Xu L., Xu Y., Perini T., Senapedis W. (2019). Dual PAK4-NAMPT Inhibition Impacts Growth and Survival, and Increases Sensitivity to DNA-Damaging Agents in Waldenstrom Macroglobulinemia. Clin. Cancer Res..

[35] Ozgencil F., Gunindi H.B., Eren G. (2024). Dual-targeted NAMPT inhibitors as a progressive strategy for cancer therapy. Bioorg. Chem..

[36] Vargas B., Boslett J., Yates N., Sluis-Cremer N. (2023). Mechanism by Which PF-3758309, a Pan Isoform Inhibitor of p21-Activated Kinases, Blocks Reactivation of HIV-1 Latency. Biomolecules.

[37] Verma A., Najahi-Missaoui W., Cummings B.S., Somanath P.R. (2020). Sterically stabilized liposomes targeting P21 (RAC1) activated kinase-1 and secreted phospholipase A2 suppress prostate cancer growth and metastasis. Oncol. Lett..

[38] Wang Y., Minden A. (2024). Inhibition of NAMPT by PAK4 Inhibitors. Int. J. Mol. Sci..

[39] Mitchell S., Zhang P., Cannon M., Beaver L., Lehman A., Harrington B., Sampath D., Byrd J.C., Lapalombella R. (2021). Anti-tumor NAMPT inhibitor, KPT-9274, mediates gender-dependent murine anemia and nephrotoxicity by regulating SIRT3-mediated SOD deacetylation. J. Hematol. Oncol..

[40] Naing A., Leong S., Pishvaian M., Razak A., Mahipal A., Berlin J., Cho D., Senapedis W., Shacham S., Kauffman M. (2017). A first in human phase 1 study of KPT-9274, a first in class dual inhibitor of PAK4 and NAMPT, in patients with advanced solid malignancies or NHL. Ann. Oncol..

[41] Wang Y., Minden A. (2022). Current Molecular Combination Therapies Used for the Treatment of Breast Cancer. Int. J. Mol. Sci..

[42] Mitchell S.R., Larkin K., Grieselhuber N.R., Lai T.-H., Cannon M., Orwick S., Sharma P., Asemelash Y., Zhang P., Goettl V.M. (2019). Selective targeting of NAMPT by KPT-9274 in acute myeloid leukemia. Blood Adv..

[43] Pant K., Richard S., Peixoto E., Yin J., Seelig D.M., Carotenuto P., Salati M., Franco B., Roberts L.R., Gradilone S.A. (2023). The NAMPT Inhibitor FK866 in Combination with Cisplatin Reduces Cholangiocarcinoma Cells Growth. Cells.

[44] Fratta S., Biniecka P., Moreno-Vargas A.J., Carmona A.T., Nahimana A., Duchosal M.A., Piacente F., Bruzzone S., Caffa I., Nencioni A. (2023). Synthesis and structure-activity relationship of new nicotinamide phosphoribosyltransferase inhibitors with antitumor activity on solid and haematological cancer. Eur. J. Med. Chem..

[45] Xu Z., Wang H., Liu H., Chen H., Jiang B. (2022). Synthesis and Evaluation of Reactive Oxygen Species Sensitive Prodrugs of a NAMPT Inhibitor FK866. Molecules.

[46] Tang H., Wang L., Wang T., Yang J., Zheng S., Tong J., Jiang S., Zhang X., Zhang K. (2023). Recent advances of targeting nicotinamide phosphoribosyltransferase (NAMPT) for cancer drug discovery. Eur. J. Med. Chem..

[47] Ramos-Alvarez I., Jensen R.T. (2018). P21-activated kinase 4 in pancreatic acinar cells is activated by numerous gastrointestinal hormones/neurotransmitters and growth factors by novel signaling, and its activation stimulates secretory/growth cascades. Am. J. Physiol. Gastrointest. Liver Physiol..

[48] Zhang J., Wang J., Guo Q., Wang Y., Zhou Y., Peng H., Cheng M., Zhao D., Li F. (2012). LCH-7749944, a novel and potent p21-activated kinase 4 inhibitor, suppresses proliferation and invasion in human gastric cancer cells. Cancer Lett..

[49] Zhuang T., Zhu J., Li Z., Lorent J., Zhao C., Dahlman-Wright K., Strömblad S. (2015). p21-activated kinase group II small compound inhibitor GNE-2861 perturbs estrogen receptor alpha signaling and restores tamoxifen-sensitivity in breast cancer cells. Oncotarget.

[50] Murray B.W., Guo C., Piraino J., Westwick J.K., Zhang C., Lamerdin J., Dagostino E., Knighton D., Loi C.-M., Zager M. (2010). Small-molecule p21-activated kinase inhibitor PF-3758309 is a potent inhibitor of oncogenic signaling and tumor growth. Proc. Natl. Acad. Sci. USA.

[51] Sampath D., Zabka T.S., Misner D.L., O’bRien T., Dragovich P.S. (2015). Inhibition of nicotinamide phosphoribosyltransferase (NAMPT) as a therapeutic strategy in cancer. Pharmacol. Ther..

[52] Al-Sanea M.M., Chilingaryan G., Abelyan N., Mamikonyan M., Gasparyan H., Hovhannisyan S., Hamdi A., Ali A.R., Selim S., Mohamed A.A.B. (2022). Combination of ligand and structure based virtual screening approaches for the discovery of potential PARP1 inhibitors. PLoS ONE.

[53] Ortiz C.L.D., Completo G.C., Nacario R.C., Nellas R.B. (2019). Potential Inhibitors of Galactofuranosyltransferase 2 (GlfT2): Molecular Docking, 3D-QSAR, and In Silico ADMETox Studies. Sci. Rep..

[54] Iman M., Saadabadi A., Davood A. (2015). Molecular docking analysis and molecular dynamics simulation study of ameltolide analogous as a sodium channel blocker. Turk. J. Chem..

[55] Waszkowycz B. (2008). Towards improving compound selection in structure-based virtual screening. Drug Discov. Today.

[56] Zhao M., Ma J., Li M., Zhang Y., Jiang B., Zhao X., Huai C., Shen L., Zhang N., He L. (2021). Cytochrome P450 Enzymes and Drug Metabolism in Humans. Int. J. Mol. Sci..

[57] Deodhar M., Al Rihani S.B., Arwood M.J., Darakjian L., Dow P., Turgeon J., Michaud V. (2020). Mechanisms of CYP450 Inhibition: Understanding Drug-Drug Interactions Due to Mechanism-Based Inhibition in Clinical Practice. Pharmaceutics.

[58] Xu T., OMZhao J., Sakamuru S., Ngan D.K., Zhang L., Yang S., Travers J., Xia M., Zhao T. (2025). Expanded Tox21 biological assay panel for the prediction of drug-induced liver injury and cardiotoxicity. Environ. Health Perspect..

[59] Kwon H.J., Lee H., Lee S., Ko W., Yun S.J., Uesawa Y., Jung J. (2025). Comparative analysis of OECD guideline data and Tox21 assays to improve reproductive and developmental toxicity prediction. Sci. Rep..

[60] Petin K., Weiss R., Müller G., Garten A., Grahnert A., Sack U., Hauschildt S. (2019). NAD metabolites interfere with proliferation and functional properties of THP-1 cells. Innate Immun..

[61] Ye J., Wu J., Liu B. (2023). Therapeutic strategies of dual-target small molecules to overcome drug resistance in cancer therapy. Biochim. Biophys. Acta Rev. Cancer.

[62] Roy R., Ria T., RoyMahaPatra D., Sk U.H. (2023). Single Inhibitors versus Dual Inhibitors: Role of HDAC in Cancer. ACS Omega.

[63] He Y., Liu H., Bian W., Liu Y., Liu X., Ma S., Zheng X., Du Z., Zhang K., Ouyang D. (2019). Molecular Interactions for the Curcumin-Polymer Complex with Enhanced Anti-Inflammatory Effects. Pharmaceutics.

[64] Zhu J., Huang Q. (2019). Nanoencapsulation of functional food ingredients. Adv. Food Nutr. Res..

[65] Martorana F., Motta G., Pavone G., Motta L., Stella S., Vitale S.R., Manzella L., Vigneri P. (2021). AKT Inhibitors: New Weapons in the Fight Against Breast Cancer?. Front. Pharmacol..

[66] Leung J.H., Leung H.W., Wang S.Y., Huang S.S., Chan A.L. (2021). Efficacy and safety of CDK4/6 and PI3K/AKT/mTOR inhibitors as second-line treatment in postmenopausal patients with hormone receptor-positive, HER-2-negative metastatic breast cancer: A network meta-analysis. Expert Opin. Drug Saf..

[67] Navas L.E., Carnero A. (2022). Nicotinamide Adenine Dinucleotide (NAD) Metabolism as a Relevant Target in Cancer. Cells.

[68] Reddy T.P., Rosato R.R., Li X., Moulder S., Piwnica-Worms H., Chang J.C. (2020). A comprehensive overview of metaplastic breast cancer: Clinical features and molecular aberrations. Breast Cancer Res..

[69] Cao L., Niu Y. (2020). Triple negative breast cancer: Special histological types and emerging therapeutic methods. Cancer Biol. Med..

[70] Royce M., Bachelot T., Villanueva C., Özgüroglu M., Azevedo S.J., Cruz F.M., Debled M., Hegg R., Toyama T., Falkson C. (2018). Everolimus Plus Endocrine Therapy for Postmenopausal Women with Estrogen Receptor-Positive, Human Epidermal Growth Factor Receptor 2-Negative Advanced Breast Cancer: A Clinical Trial. JAMA Oncol..

[71] Mishra R., Patel H., Alanazi S., Kilroy M.K., Garrett J.T. (2021). PI3K Inhibitors in Cancer: Clinical Implications and Adverse Effects. Int. J. Mol. Sci..

[72] Won K., Spruck C. (2020). Triplenegative breast cancer therapy: Current and future perspectives (Review). Int. J. Oncol..

[73] Bedard P.L., Hyman D.M., Davids M.S., Siu L.L. (2020). Small molecules, big impact: 20 years of targeted therapy in oncology. Lancet.

[74] Lima Z.S., Ghadamzadeh M., Arashloo F.T., Amjad G., Ebadi M.R., Younesi L. (2019). Recent advances of therapeutic targets based on the molecular signature in breast cancer: Genetic mutations and implications for current treatment paradigms. J. Hematol. Oncol..

[75] Indini A., Fiorilla I., Ponzone L., Calautti E., Audrito V. (2022). NAD/NAMPT and mTOR Pathways in Melanoma: Drivers of Drug Resistance and Prospective Therapeutic Targets. Int. J. Mol. Sci..

[76] Mir M., Khan H., Mehraj U., Nisar S., Bhat B., Wani N. (2020). Targeting Different Pathways Using Novel Combination Therapy in Triple Negative Breast Cancer. Curr. Cancer Drug Targets.

[77] Jhan J.R., Andrechek E.R. (2017). Triple-negative breast cancer and the potential for targeted therapy. Pharmacogenomics.

[78] Fang G., Chen H., Cheng Z., Tang Z., Wan Y. (2023). Azaindole derivatives as potential kinase inhibitors and their SARs elucidation. Eur. J. Med. Chem..

[79] DeAngelo D.J., Radia D.H., George T.I., Robinson W.A., Quiery A.T., Drummond M.W., Bose P., Hexner E.O., Winton E.F., Horny H.-P. (2021). Safety and efficacy of avapritinib in advanced systemic mastocytosis: The phase 1 EXPLORER trial. Nat. Med..

[80] Xu H., Zhang Y., Liu J., Cui J., Gan Y., Wu Z., Chang Y., Sui R., Chen Y., Shi J. (2022). UM-164, a Dual Inhibitor of c-Src and p38 MAPK, Suppresses Proliferation of Glioma by Reducing YAP Activity. Cancers.

[81] Knippler C.M., Saji M., Rajan N., Porter K., La Perle K.M.D., Ringel M.D. (2019). MAPK- and AKT-activated thyroid cancers are sensitive to group I PAK inhibition. Endocr. Relat. Cancer.

[82] Lu H., Liu S., Zhang G., Wu B., Zhu Y., Frederick D.T., Hu Y., Zhong W., Randell S., Sadek N. (2017). PAK signalling drives acquired drug resistance to MAPK inhibitors in BRAF-mutant melanomas. Nature.

[83] Garten A., Schuster S., Penke M., Gorski T., de Giorgis T., Kiess W. (2015). Physiological and pathophysiological roles of NAMPT and NAD metabolism. Nat. Rev. Endocrinol..

[84] Rudolph J., Crawford J.J., Hoeflich K.P., Wang W. (2015). Inhibitors of p21-activated kinases (PAKs). J. Med. Chem..

[85] Shah M., Wedam S., Cheng J., Fiero M.H., Xia H., Li F., Fan J., Zhang X., Yu J., Song P. (2021). FDA Approval Summary: Tucatinib for the Treatment of Patients with Advanced or Metastatic HER2-positive Breast Cancer. Clin. Cancer Res..

[86] Mogwera K.S., Chibale K., Arendse L.B. (2023). Developing kinase inhibitors for malaria: An opportunity or liability?. Trends Parasitol..

[87] Wang Y., He X., Li C., Ma Y., Xue W., Hu B., Wang J., Zhang T., Zhang F. (2020). Carvedilol serves as a novel CYP1B1 inhibitor, a systematic drug repurposing approach through structure-based virtual screening and experimental verification. Eur. J. Med. Chem..

[88] Pushpakom S., Iorio F., Eyers P.A., Escott K.J., Hopper S., Wells A., Doig A., Guilliams T., Latimer J., McNamee C. (2019). Drug repurposing: Progress, challenges and recommendations. Nat. Rev. Drug Discov..

[89] Wu K.-J., Zhong H.-J., Li G., Liu C., Wang H.-M.D., Ma D.-L., Leung C.-H. (2018). Structure-based identification of a NEDD8-activating enzyme inhibitor via drug repurposing. Eur. J. Med. Chem..

[90] Khan O.U.R., Khan B.A., Hamdani S.S., Jalil S., Sidhom P.A., Ibrahim K.E., Abalkhail T., Iqbal J., Tallima H., Shoeib T. (2025). Design, Synthesis, Biological Evaluation, and In Silico Study of Tetrahydropyridines as Prospective Monoamine Oxidase Inhibitors. ChemistryOpen.

[91] Morak-Młodawska B., Jeleń M., Martula E., Korlacki R. (2023). Study of Lipophilicity and ADME Properties of 1,9-Diazaphenothiazines with Anticancer Action. Int. J. Mol. Sci..

[92] Wang Y., Li D., Lin H., Jiang S., Han L., Hou S., Lin S., Cheng Z., Bian W., Zhang X. (2020). Enhanced oral bioavailability and bioefficacy of phloretin using mixed polymeric modified self-nanoemulsions. Food Sci. Nutr..

[93] Beheshtirouy S., Mirzaei F., Eyvazi S., Tarhriz V. (2021). Recent Advances in Therapeutic Peptides for Breast Cancer Treatment. Curr. Protein Pept. Sci..

[94] Rudolph J., Murray L.J., Ndubaku C.O., O’Brien T., Blackwood E., Wang W., Aliagas I., Gazzard L., Crawford J.J., Drobnick J. (2016). Chemically Diverse Group I p21-Activated Kinase (PAK) Inhibitors Impart Acute Cardiovascular Toxicity with a Narrow Therapeutic Window. J. Med. Chem..

