# Supplementary material for: Computational Identification of New Dual PAK4 and NAMPT Inhibitors
Source: Int J Mol Sci. 2026 Aug 28;27(17):7706. doi: 10.3390/ijms27177706 (PMC13565989; doi:10.3390/ijms27177706)
Supplement: Supplementary file 1 [file ijms-27-07706-s001.zip › ijms-4430510-supplementary.pdf]

Supplementary Table S1. Top 48 Candidate Compounds

	Smiles	PUBCHEM_COMPOUND_CID	Drug name	Top S-NAMPT	S-PAK4
1	C1CN(CCC1(F)F)C(=O)C2=CC=C(C=C2)C3=CC(=C4C(=C3)C=C(O4)CNC(=O)/C=C/C5=CN=C(C=C5)N)C6=CC=C(C=C6)F	117779453	KPT-9274	-7.25	-7.5
2	CN(C)C/C=C/C(=O)NC1=C(C=C2C(=C1)C(=NC=N2)NC3=CC(=C(C=C3)F)Cl)O[C@H]4CCOC4	10184653	Afatinib	-8.67	-7.18
3	CC1=C(C=CC(=C1)NC2=NC=NC3=C2C=C(C=C3)NC4=NC(CO4)(C)C)OC5=CC6=NC=NN6C=C5	51039094	Tucatinib	-8.66	-7.89
4	CN(C)CC1=CN(N=C1C2=CC=CC=C2)C3=NC(=NC=C3)NC4=C(C=C(C(=C4)NC(=O)C=C)N5CCOCC5)OC	121269225	Lazertinib	-8.34	-7.15
5	CN(C)CCN(C)C1=CC(=C(C=C1NC(=O)C=C)NC2=NC=CC(=N2)C3=CNC4=CC=CC=C43)OC	71496460	Osimertinib	-8.32	-7.01
6	COC1=C(C=CC(=C1)C2CN(C2)C(=O)C3=NC=CC(=C3)COCC(CO)O)OCC4=CC=C(C=C4)C5CC5	59633972	JTE-952	-8.15	-7.43
7	CC1CCC2CC(C(=CC=CC=CC(CC(C(=O)C(C(C(=CC(C(=O)CC(OC(=O)C3CCCCN3C(=O)C(=O)C1(O2)O)C(C)CC4CCC(C(C4)OC)OC(=O)C(C)(CO)CO)C)C)O)OC)C)C)C)OC	148191	Temsirolimus	-7.93	-7.50
8	C[C@](C1=CC=C(C=C1)F)(C2=CN=C(N=C2)N3CCN(CC3)C4=NC=NN5C4=CC(=C5)C6=CN(N=C6)C)N	118023034	Avapritinib	-7.90	-7.15
9	CC1=C(C=C(C=C1)NC(=O)C2=CC(=CC=C2)C(F)(F)F)NC(=O)C3=CN=C(S3)NC4=CC(=NC(=N4)C)N5CCN(CC5)CCO	11714353	UM-164	-7.85	-7.56
10	CS(=O)(=O)C1=C(C=C(C=C1)C(F)(F)F)NC(=O)NC2=CN=C(N=C2)OC3=CC=C(C=C3)C4=C(N=CC(=C4)Cl)N	78322573	ONO-7579	-7.85	-8.41
11	CC1=C(C=C(C=C1)NC2=NC=C(C(=N2)NC)C(=O)NC3=C(C=CC(=C3)NC(=O)C4=CC(=C(C(=C4)OC)OC)OC)C)NC(=O)C=C	122633900	CHMFL-BMX-078	-7.84	-8.15
12	C=CC(=O)N1CCC(CC1)C2CCNC3=C(C(=NN23)C4=CC=C(C=C4)OC5=CC=CC=C5)C(=O)N	135905454	Zanubrutinib	-7.77	-7.03
13	CC(C)(C1=NC(=CC=C1)N2C3=NC(=NC=C3C(=O)N2CC=C)NC4=CC=C(C=C4)N5CCN(CC5)C)O	24856436	Adavosertib (MK-1775)	-7.77	-7.40
14	CC1=C(C=C(C=C1)C(=O)NC2=CC(=CC(=C2)C(F)(F)F)N3C=C(N=C3)C)NC4=NC=CC(=N4)C5=CN=CC=C5	644241	Nilotinib	-7.74	-7.45
15	CC1=C(C=C(C=C1)C(=O)NC2=CC(=C(C=C2)CN3CCN(CC3)C)C(F)(F)F)C#CC4=CN=C5N4N=CC=C5	24826799	Ponatinib	-7.74	-7.90

16	CC1=C(C=C(C=N1)NC(=O)C2=CC(=C(C=C2)CN3CCN(CC3)C)C(F)(F)F)NC4=NC=CC(=N4)C5=CN=CC=C5	46848036	Flumatinib	-7.71	-7.36
17	CC1=C(C=C(C=C1)NC(=O)C2=CC=C(C=C2)CN3CCN(CC3)C)NC4=NC=CC(=N4)C5=CN=CC=C5	5291	Imatinib	-7.69	-7.12
18	CC1=C(C=CC=C1C(F)(F)F)CN2C(=NC3=C(C=C(C=C32)N4CCOCC4)C(=O)O)C	56949517	GSK2636771	-7.66	-7.05
19	C[C@@H]1CC[C@H]2C[C@@H](C(=C/C=C/C=C/[C@H](C[C@H](C(=O)[C@@H]([C@@H](C(=C[C@H](C(=O)C[C@H](OC(=O)[C@@H]3CCCCN3C(=O)C(=O)[C@@]1(O2)O)[C@H](C)C[C@@H]4CC[C@H]([C@@H](C4)OC)OC(=O)C(C)(CO)CO)C)C)O)OC)C)C)C)OC	54608508	Torisel, NSC-683864	-7.64	-7.43
20	CCC1=C(N=C(C(=N1)C(=O)N)NC2=CC=C(C=C2)N3CCC(CC3)N4CCN(CC4)C)O[C@@H]5CCN(C5)C(=O)C=C	71667668	Naquotinib	-7.64	-7.72
21	CN1CCC(CC1)COC2=CN=C(N=C2)C3=CC=CC(=C3)CN4C(=O)C=CC(=N4)C5=CC=CC(=C5)C#N	25171648	Tepotinib (EMD-1214063)	-7.64	-7.26
22	CN(C)C1CCN(CC1)C(=O)C2=CC=C(C=C2)NC(=O)NC3=CC=C(C=C3)C4=NC(=NC(=N4)N5CCOCC5)N6CCOCC6	44516953	Gedatolisib（PKI-587; PF-05212384）	-7.62	-7.74
23	CC(C)C[C@@H](C(=O)NCCOCCOCCOCCNC(=O)C1=CC=C(C=C1)C2=NC(=CS2)CNC3=NC=CC(=N3)NCC(C)C)NC(=O)[C@H]([C@@H](CC4=CC=CC=C4)N)O	139600269	139600269	-7.61	-8.58
24	CCCNC1=NC(=NC=C1C#CCCNC(=O)[C@H](C)N(C)C(=O)/C=C/CN(C)C)NC2=CC=C(C=C2)C#N	90052320	FLT3-IN-1<br>FF-10101	-7.61	-9.22
25	COC1=CC=CC(=C1)C2=CN(C3=NC=NC(=C23)N)C4=CC=C(C=C4)CCN5CCC(CC5)O	5311381	234772-64-6<br>（CGP77675）	-7.60	-7.39
26	CN1C=C(C2=CC=CC=C21)C3=NC(=NC=C3)NC4=C(N=C(C(=C4)NC(=O)C=C)N(C)CCN(C)C)OCC(F)(F)F	118861389	Alflutinib	-7.59	-7.36
27	CC1(C2=C(CN1C(=O)N[C@H](CN(C)C)C3=CC=CC=C3)C(=NN2)NC(=O)C4=CC=C(C=C4)NC(=O)C=C)C	121443990	YKL-5-124	-7.59	-7.21
28	CCOC1=C(C=C2C(=C1)N=CC(=C2NC3=CC(=C(C=C3)OCC4=CC=CC=N4)Cl)C#N)NC(=O)/C=C/CN(C)C	9915743	Neratinib	-7.58	-7.39
29	C[C@@H]1CC[C@H]2C[C@@H](/C(=C/C=C/C=C/[C@H](C[C@H](C(=O)[C@@H]([C@@H](/C(=C/[C@H](C(=O)C[C@H](OC(=O)[C@@H]3CCCCN3C(=O)C(=O)[C@@]1(O2)O)[C@H](C)C[C@@H]4CC[C@H]([C@@H](C4)OC)OCCO)C)/C)O)OC)C)C)/C)OC	6442177	Everolimus	-7.55	-8.05
30	CC1=C(C=C(C=C1)NC(=O)C2=CC(=C(C=C2)CN3CC[C@@H](C3)N(C)C)C(F)(F)F)NC4=NC=CC(=N4)C5=CN=CN=C5	11387605	Bafetinib	-7.54	-7.10
31	CNC(=O)C1=C(C=C(C=C1)C2=NN3C(=CN=C3N=C2)CC4=CC5=C(C=C4)N=CC=C5)F	25145656	Capmatinib	-7.52	-7.07
32	CC(C)NS(=O)(=O)C1=CC=CC=C1NC2=NC(=NC3=C2CCN3)NC4=C(C=C(C=C4)N5CCC(CC5)N6CCN(CC6)C)OC	89860551	Conteltinib	-7.49	-8.25

33	COC1=C(C=CC2=C3NCCN3C(=NC(=O)C4=CN=C(N=C4)N)N=C21)OCCCN5CCOCC5	135565596	Copanlisib(BAY 80-6946)	-7.46	-7.23
34	COCCOC1=CC=C(C=C1)NC2=NC=C(C(=N2)NC3=CC(=CC=C3)NC(=O)C=C)F	59174488	Spebrutinib	-7.46	-7.36
35	CNC(=O)CCC1=CC(=NN1C2=CC=C(C=C2)N3CCNCC3)C4=CC=C(C=C4)C5=CC=C(C=C5)C(F)(F)F	118986645	ILK-IN-2 (OSU-T315 analog)	-7.43	-7.76
36	CCCCCCCCCCCCC/C=C/[C@H]([C@H](CO)NC(=O)C)O	5497136	C2 Ceramid	-7.43	-7.71
37	CN1CCN(CC1)C2=CC(=C(C=C2)C(=O)NC3=NNC4=C3C=C(C=C4)CC5=CC(=CC(=C5)F)F)NC6CCOCC6	25141092	Entrectinib	-7.42	-7.06
38	COC1=CC(=CC(=C1)C#CC2=NN(C3=NC=NC(=C23)N)[C@H]4CCN(C4)C(=O)C=C)OC	71621331	Futibatinib	-7.41	-7.38
39	CC1=CN=C(N=C1NC2=CC(=CC=C2)S(=O)(=O)NC(C)(C)C)NC3=CC=C(C=C3)OCCN4CCCC4	16722836	Fedratinib	-7.41	-7.18
40	CNC(=O)C1=CC=C(C=C1)NC2=NC=C3CN(CCC3=N2)C4=C(C=CC(=C4)NC(=O)C5=NC=CC(=C5)C(F)(F)F)Cl	67328148	Debio 0617B	-7.38	-8.03
41	CCC1=C(N=C(C(=N1)C(=O)N)NC2=CC(=C(C=C2)N3CCC(CC3)N4CCN(CC4)C)OC)NC5CCOCC5	49803313	Gilteritinib (ASP2215)	-7.35	-7.88
42	CC(C)OC1=CC(=CC2=C1C(=NC=N2)NC3=C4C(=NC=C3Cl)OCO4)OCCN5CCN(CC5)C(=O)C	9893171	AZD0424	-7.32	-7.24
43	CCCCCCCCCCCCCCCCCCOCC(COP(=O)([O-])OCC[N+](C)(C)C)OC	1392	Edelfosine	-7.31	-7.44
44	CC1=CC2=C(N1)C=CC(=C2)OC3=NC(=NC=C3)NC4=CC=CC(=C4)CS(=O)(=O)NCCN(C)C	52920501	Sulfatinib	-7.29	-7.48
45	CC(C)(C)C1=NN(C(=C1)NC(=O)NC2=C(C=C(C=C2)OC3=CC(=NC=C3)C(=O)NC)F)C4=CC5=C(C=C4)N=CC=C5	25066467	Rebastinib	-7.28	-7.79
46	CC(C)NC(=O)C1CCC(CC1)N2C3=C(C=CC(=C3)CN4CCC(CC4)C(C)(C)O)N=C2NC(=O)C5=CC=C(C=C5)F	57345941	TSR-011	-7.27	-7.96
47	CC1=C(C(=CC=C1)Cl)NC(=O)C2=CN=C(S2)NC3=CC(=NC(=N3)C)N4CCN(CC4)CCO	3062316	Dasatinib	-7.25	-7.00
48	CCN1CCN(CC1)CC2=C(C=C(C=C2)NC(=O)C3=CC(=C(C=C3)C)OC4=C5C=CNC5=NC=C4)C(F)(F)F	53340664	NG25	-7.16	-7.78
